# A New APEH Cluster with Antioxidant Functions in the Antarctic Hemoglobinless Icefish *Chionodraco hamatus*

**DOI:** 10.1371/journal.pone.0125594

**Published:** 2015-05-06

**Authors:** Alessia Riccio, Marta Gogliettino, Gianna Palmieri, Marco Balestrieri, Angelo Facchiano, Mosè Rossi, Stefania Palumbo, Giuseppe Monti, Ennio Cocca

**Affiliations:** 1 National Research Council, Institute of Biosciences and BioResources (CNR-IBBR), Napoli, Italy; 2 National Research Council, Institute of Food Sciences (CNR-ISA), Avellino, Italy; 3 Ittica Literno srl, Caserta, Italy; Institute of Hydrobiology, Chinese Academy of Sciences, CHINA

## Abstract

Acylpeptide hydrolase (APEH) is a ubiquitous cytosolic protease that plays an important role in the detoxification of oxidised proteins. In this work, to further explore the physiological role of this enzyme, two *apeh* cDNAs were isolated from the *Chionodraco hamatus* icefish, which lives in the highly oxygenated Antarctic marine environment. The encoded proteins (APEH-1*_Ch_* and APEH-2_*Ch*_) were characterised in comparison with the uniquely expressed isoform from the temperate fish *Dicentrarchus labrax* (APEH-1_*Dl*_) and the two APEHs from the red-blooded Antarctic fish *Trematomus bernacchii* (APEH-1_*Tb*_ and APEH-2_*Tb*_). Homology modelling and kinetic characterisation of the APEH isoforms provided new insights into their structure/function properties. APEH-2 isoforms were the only ones capable of hydrolysing oxidised proteins, with APEH-2_*Ch*_ being more efficient than APEH-2_*Tb*_ at this specific function. Therefore, this ability of APEH-2 isoforms is the result of an evolutionary adaptation due to the pressure of a richly oxygenated environment. The lack of expression of APEH-2 in the tissues of the temperate fish used as the controls further supported this hypothesis. In addition, analysis of gene expression showed a significant discrepancy between the levels of transcripts and those of proteins, especially for *apeh-2* genes, which suggests the presence of post-transcriptional regulation mechanisms, triggered by cold-induced oxidative stress, that produce high basal levels of APEH-2 mRNA as a reserve that is ready to use in case of the accumulation of oxidised proteins.

## Introduction

The aerobic metabolism leads to the production of noxious reactive oxygen species (ROS) produced by the reduction of molecular oxygen to water. For this reason, eukaryotic cells have developed special defence systems that are involved in multistep processes comprising several low-molecular weight free radical scavengers (glutathione, beta-carotene and vitamins), and a number of specific enzymes such as superoxide dismutase (SOD), catalase and glutathione peroxidase [1,2]. Oxidative tissue damage induces lipid peroxidation, DNA damage, protein degradation and cell death [1–4] and occurs when antioxidant defences are overcome by cellular production of ROS. Investigations on vertebrate species indicate that at least 90% of ROS generated in cells originates in mitochondria [1,5].

Fish comprise approximately two thirds of vertebrates and constitute a biological group with unique molecular adaptations, which throughout evolution enabled them to successfully inhabit marine environments. An example of a typical physiological adaptation is the development of mitochondria, nuclei and other organelles in piscine red cells, in contrast to those of mammals, which lack these cell structures [6–8]. Therefore, fish erythrocytes have been demonstrated to provide an excellent model for studies on ROS and antioxidant systems.

In the last years, a growing interest has been addressed to the study of the antioxidant defence systems in fish inhabiting permanently cold marine environments, such as polar oceans [9], to better understand the oxidative stress processes. In seawater, the solubility and the concentration of oxygen increase considerably by 40% between 15°C and 0°C; therefore, very cold Antarctic waters (-1.9–2°C) produce increased tissue oxygenation with consequential major oxidative stress levels in polar, compared to temperate, ectotherms. Moreover, a common feature of cold adaptation in polar fish is a high density of mitochondria [8], which leads to ROS accumulation under stress, rendering these animal species prone to elevated oxidative injury. This exposure is further aggravated by an increased unsaturation of fatty acids in fish from Antarctic seas, which leads to a potential enhancement of lipid peroxidation induced by ROS [8]. For all these reasons, the Antarctic fish fauna requires a really strong defence system against the oxidative stress that requires appropriate biochemical strategies. It has been shown that these fishes, despite the relatively low activity of some antioxidant enzymes, compensate for the threat of increased oxidative damage by maintaining high levels of glutathione, vitamin E and other low molecular weight scavengers in the plasma or incorporated into the membranes [10].

Recently, acylpeptide hydrolase (APEH), which was first identified as oxidised protein hydrolase (OPH), has been hypothesised to participate in ROS detoxification as a member of the phase 3 antioxidant enzymes [11], which are involved in the elimination of irreversibly denatured proteins. Confirming this hypothesis, we showed in a previous study [12] that APEH plays a key role in the degradation of oxidised and cytotoxic proteins specifically in Antarctic fish.

APEH is a serine peptidase able to catalyse the removal of Nα-acetylated amino acids from blocked peptides and it belongs to the prolyl oligopeptidase (POP) family (clan SC, family S9). It is a crucial regulator of N-terminally acetylated proteins in eukaryal, bacterial and archaeal cells [13–15] but its biological role has not yet been completely elucidated. The only 3D structure reported to date is related to an APEH from the hyperthermophilic microorganism *Aeropyrum pernix*, which exhibits a peptidase domain with a α/β-hydrolase fold, and an unusual β-propeller, which covers the catalytic triad [14–16] and protects large, structured peptides and proteins from proteolysis by the active site. Unlike the tetrameric mammalian APEH, the archaeal counterpart shows a homodimeric structure which shows the typical structural features of the POP family [17].

We identified the first APEHs from the Antarctic fish *Trematomus bernacchii* [12]. Specifically, we demonstrated the presence of two active APEH isoforms (APEH-1_*Tb*_ and APEH-2_*Tb*_) belonging to different phylogenetic clusters and exhibiting distinct molecular and temperature–kinetic behaviours. Interestingly, APEH-1_*Tb*_ showed the typical enzymatic properties of APEHs studied so far, whereas APEH-2_*Tb*_ appeared to be a bifunctional enzyme that exhibits exopeptidase activity towards N-acyl peptides and efficient endoprotease activity towards oxidised proteins, indicating that this isoform plays a key homeostatic role in sustaining the protective systems required to counteract cold-induced oxidative stress [12].

In this context, the purpose of this work was to further investigate the role of APEH as an antioxidant enzyme by comparing members of this protein family from fish living in environmental stress conditions [3] or in temperate seawaters. Therefore, we focused our attention on *Chionodraco hamatus*, an Antarctic fish belonging to the family of *Channichthydae* (also known as icefish), and on *Dicentrarchus labrax*, the European sea bass that belongs to the family of *Moronidae*. Icefish are one of the better models for understanding the effects of the oxidative stress on living species; in fact, they are a unique example of an adult vertebrate that lacks haemoglobin and functionally active erythrocytes [18]. To compensate for this, icefish have developed peculiar phenotypic traits such as improved skin vascularisation, cutaneous uptake of molecular oxygen, a hypertrophic heart, increased blood volume, enlarged vessel lumina, low blood viscosity and a reduced metabolic rate. [8,19–21].

In this study, two new APEHs from *C*. *hamatus* (APEH-1_*Ch*_ and APEH-2_*Ch*_) were functionally/structurally characterised and compared to the unique APEH isoform (APEH-1_*Dl*_) found in the temperate *D*. *labrax*. The cDNAs of the corresponding coding genes was isolated to explore their function in the physiology of Antarctic *vs* temperate fish and their involvement in the response to environmental oxidative stress. Our investigations strongly confirm the distinct functional/structural properties of the members belonging to cluster 1 (C1) and cluster 2 (C2) of the APEH family. Specifically, APEH-2_*Ch*_ shows high efficiency at hydrolysing oxidised proteins and for this reason, it may represent a biological innovation to combat the specific oxidative stress conditions.

## Materials and Methods

### Ethical procedures

The sample collection and experimental research conducted on the animals utilized in this study were according to the law on activities and environmental protection in Antarctica approved by the Ministry of Education, Universities and Research of the Republic of Italy (MIUR), to comply with the "Protocol on Environmental Protection to the Antarctic Treaty", Annex II, art.3. All procedures, including euthanasia, were reviewed and approved by MIUR and performed in accordance with the European Communities Council Directive of 24 November 1986 (86/609/EEC).

### Animal sampling

*C*. *hamatus* specimens were fished in the vicinity of Mario Zucchelli Station, along the coast of Terra Nova Bay (74′42°S, 164′07°E), Antarctica, during the Italian XXV and XXVII expeditions (December 2009—January 2010 and December 2011—January 2012, respectively). They were held in running seawater at -2°C to +1°C until tissue sampling. *D*. *labrax* specimens were collected at a fish farm. The water temperature was controlled at about 18°C. The animals were anesthetized with tricaine methanesulphonate (MS222, 300 mg/liter) for at least 30 min before being killed by truncation of the spinal cord. Tissues were dissected from adult specimens, and frozen immediately in liquid nitrogen. Blood was drawn from the caudal vein with heparinized syringes. Blood cells (BC) were collected by centrifugation at 3000 g for 5 min, washed in 1.7% NaCl, and then frozen in liquid nitrogen. Tissues and cells were stored at -80°C until use.

### Cloning and sequence analysis

Total RNAs were isolated from liver of *C*. *hamatus* and *D*. *labrax* according to the PureLink RNA Mini Kit (Life Technologies) protocol, with an on-column DNase I digestion. RNA concentrations were determined with a Qubit Fluorometer (Invitrogen). RNAs were then reverse transcribed with the SuperScript VILO MasterMix (Invitrogen). The *apeh-1 and apeh-2* cDNAs were amplified by PCR with oligonucleotides designed on the basis of the homologues sequences from *T*. *bernacchii* (KC626077 and KC626078) [11], *Takifugu rubripes* (XM_003963278.1 and XM_003963026.1), *Oreochromis niloticus* (XM_003448291.1 and XM_003444841.1), Oryzias latipes (XR_177409.1 and XM_004070616.1), *Danio rerio* (NM_198869.1 and NM_001040347.1), and of BLAST analysis of a notothenioid SRA library (SRS255209) [22]. The primers used were as follows: APEH-1_*Ch*_for, 5′-ATGAACTCACAGGTGGTGACC-3′, and APEH-1_*Ch*_rev, 5′-TCACTTCCACAAGTGTTGAATTATCCA-3′, for *apeh-1*_*Ch*_; APEH-2_*Ch*_for, 5′-ATGGAGCCCAGCCTGGT-3′, and APEH-2_*Ch*_rev, 5′-CTGTGTTGAGGAAGCAGTCGG-3, for *apeh-2*_*Ch*_; APEH-1_*Dl*_for, 5’-TGATGGTCGTCAGCTGCT-3’; and APEH-1_*Dl*_rev, 5’- CAGTCTGGGATGTCCGTG-3’, for *apeh-1*_*Dl*_; APEH-2_*Dl*_for, 5’- ATTTTGTCCCTAATTGGTCAGATC-3’, and APEH-2_*Dl*_rev, 5’-AGGAAGCAGTCAGACTGAG-3’, for *apeh-2*_*Dl*_. The amplifications were performed as follows: 94°C for 2 min, 40 cycles of 94°C (30 s), 58–60°C (30 s), and 72°C (2–2.5 min), and a final extension at 72°C for 10 min, as described in details previously [12]. The PCR products were analyzed on 1% agarose gel, purified with the StrataPrep DNA Gel Extraction Kit (Stratagene), and cloned with the StrataClone PCR Cloning kit (Stratagene). The sequences of positive clones were determined with an ABI PRISM 3100 automated sequencer at PRIMM (Milan, Italy). The sequences were edited and analyzed with the CLCMAIN WORKBENCH 6.9 program (CLC bio, 2013).

The cDNA sequences described in this study were deposited in the GenBank database under the accession numbers **KF981865** (*apeh-1*_*Ch*_), **KF981866** (*apeh-2*_*Ch*_), **KJ859684** (*apeh-1*_*Dl*_) and **KJ859685** (*apeh-2*_*Dl*_).

### Phylogenetic analysis

Phylogenetic and molecular evolutionary analyses were conducted using MEGA version 6 [23]. Multiple amino acid sequences alignment of APEH chains was obtained by use of the MUSCLE program. The alignment was tested by “Find Best DNA/Protein Models (ML)” option to search the most appropriate evolutionary model. Maximum likelihood analysis was performed with the “Construct/Test Maximum Likelihood Tree (ML)” option, using the parameters indicated by the evolutionary model (substitution model JTT+G) and the bootstrap test with 100 replicates. Finally, a phylogenetic tree with the best bootstrap consensus was obtained.

### Molecular modelling and structural analysis

The amino acid sequences of APEH-1_*Ch*_ and APEH-2_*Ch*_ were used to create molecular models based on the template structure of APEH from *Aeropyrum pernix* (Protein Data Bank code: 1VE6). To model the two proteins, we used a well-assessed strategy developed in our laboratory [24,25], which applies the comparative modelling approach by means of bioinformatics tools to search for templates, align target and template sequences, model the target, analyze the quality of the models. If needed, we iterate from the alignment step to the end, to improve the alignment and obtain satisfactory results. The search for similarity with proteins of known 3D structures is performed with BLAST. The alignment of target and template sequences obtained from the BLAST result is improved by extending to the complete sequence, and with subtle assessment of gaps by considering secondary structure position and any known feature at sequence level. This means that alternative alignments could be generated and then used for the modelling. For each alignment, 10 models are generated with MODELLER v9.10 (http://www.salilab.org). Validation procedure consists of the analysis of the models in terms of structural properties and quality, by using PROCHECK, PROSA, and visual inspection with molecular graphics tools. Finally, for each protein the best-quality model is selected for further investigations.

The analysis for the presence of H-bonds has been performed by using the HBplus tool [26], while salt bridges have been evaluated with in-house developed software essentially based on the same parameters used for the online tool ESBRI [27].

### Expression analysis

Total RNAs from several tissues (Blood Cells-BC, heart, brain, head kidney, spleen, gills, ovary, testis and liver) of three adult specimens of *C*. *hamatus* and *D*. *labrax*, respectively, were isolated and reverse transcribed under the conditions described in “Cloning and sequence analysis”. *D*. *labrax* ovaries were not analyzed because the animals sampled from the fish farm were all males. A total of 100 ng of cDNA and its dilution series were used as a template for quantitative real-time PCR to calculate the efficiency of the primers. The assays were performed on an iCycler iQTM (Bio-Rad) with 300 nM gene-specific primers, EvaGreen qPCR Mastermix (abm), and the following PCR conditions: one cycle at 95°C for 10 min, and 40 cycles at 95°C for 15 s, 60°C for 30 s, and 72°C for 30 s. The expression level of the *β*-actin gene was used as an internal control for normalization. Raw cycle threshold values (Ct values) obtained for *apeh-1* and *apeh-2* genes (target genes) were compared with the Ct value obtained for *β*-actin transcript levels (reference gene), as described in detail previously [12]. All data are expressed as mean expression fold from triplicates and the final graphical data were derived from the following equation: R = (E_target_)^ΔCt_target (control—sample)^/(E_ref_)^ΔCt_ref (control—sample)^ [28]. The Universal Probe Library Assay Design Center (http://lifescience.roche.com/shop/CategoryDisplay?catalogId=10001&tab=Assay+Design+Center&identifier=Universal+Probe+Library&langId=-1#tab-3) was used for the design of primers. The primers used in the assay are listed in Table 1.

**Table 1:** Primers used in the real-time RT-PCR assay.

Primer	Sequence	Tm (°C)	Amplicon length (bp)	Efficiency
***β*-actin*Ch*_dir**	5′-ATGTTCGAGACCTTCAACACC-3′	59.5		
			74	0.959
***β*-actin*Ch*_rev**	5′-CGACCAGAGGCGTACAGG-3′	61.4		
***β*-actin*Dl*_dir**	5′-ATGTTCGAGACCTTCAACACC-3′	59.5		
			74	1.107
***β*-actin*Dl*_rev**	5′- CGACCAGAGGCATACAGG-3′	58.7		
**APEH-1*Ch*_dir**	5′-GGTCAACTATCGAGGCTCCACTG-3′	63.5		
			62	1.102
**APEH-1*Ch*_rev**	5′-CGTTGCCAGGTAATGAGAGG-3′	59.8		
**APEH-1*Dl*_dir**	5′-CTGGTGAACTATCGTGGCTCT-3′	60.8		
			76	1.104
**APEH-1*Dl*_rev**	5′-CCTGGGAGCCTACATTGC-3′	59.3		
**APEH-2*Ch*_dir**	5′-AGTCTGCAGCAGTTGGACCT-3′	62.9		
			65	0.922
**APEH-2*Ch*_rev**	5′-GGTCTGTTGACAACCTCCAG-3′	59.6		
**APEH-2*Dl*_dir**	5′-GGATGGACAACACCCTTGAC-3′	59.9		
			61	1.054
**APEH-2*Dl*_rev**	5′-AAACCTCCATGGGAACCAC-3′	58.9		

### APEH enzyme preparation

In order to characterize the APEH proteins from the functional point of view, an optimized purification procedure was specifically applied for each enzyme due to the diversity of the features of the individual enzymes. The APEH active fractions, recovered after each purification step, were detected by measuring the proteolytic enzyme activity with the specific fluorogenic substrate Ac-Met-AMC (Acetyl-Methionine-7-amino-4-methylcoumarin).

#### a) APEH-1_Ch_ and APEH-2_Ch_ from *C*. *hamatus*

Lysis of the erythrocytes-like cells was carried out by incubation in hypotonic solution (25 mM Tris/HCl, pH 7.5) for 30 min on ice. The ‘soluble fraction’ was obtained by centrifugation of the lysate at 9200 *g* for 40 min at 4°C.

The supernatant was loaded onto a DEAE Sepharose Fast Flow column connected to an AKTA FPLC system (Amersham Biosciences), previously equilibrated in 25 mM Tris/HCl (pH 6.8) (buffer A). Bound proteins were eluted with an ionic strength gradient from 0 to 1 M NaCl in buffer A at a flow rate of 1 ml min^-1^. Two active peaks (corresponding to APEH-1_*Ch*_ and APEH-2_*Ch*_) were eluted, dialyzed against 25 mM sodium acetate, pH 5.5 (buffer A) and then separately applied to Mono S PC 1.6/5 column, connected to a SMART System (Pharmacia). The APEH active fractions were eluted with a linear gradient (0–100%) of 1 M NaCl in buffer A at a flow rate of 0.1 ml min^-1^.

At this stage of purification, two different chromatographic steps were applied. The APEH-1 active fractions, recovered from Mono S column, were pooled, dialyzed against 25 mM Tris/HCl and 1 M ammonium sulfate (pH 7.5) (buffer A), and then applied to a Phenyl Sepharose column (1.6 x 2.5 cm) (Amersham Biosciences) connected to an AKTA FPLC system equilibrated with the same buffer. Bound proteins were eluted with a linear gradient (0–100%) of 25 mM Tris/HCl (pH 7.5) (buffer B) at a flow rate of 1 ml min^-1^.

The APEH-2 active fractions, recovered from Mono S column, were pooled, dialyzed against 25 mM Tris/HCl (pH 7.5), loaded onto a Superdex 200 PC 3.2/30 column (Pharmacia Biotech), connected to a SMART System, and eluted with 25 mM Tris/HCl, pH 7.5 and 50 mM NaCl at a flow rate of 0.1 ml min^-1^.

The purified APEH proteases were stored in 25 mM Tris/HCl, pH 7.5 and 5% glycerol.

#### b) APEH-1_Dl_ from *D*. *labrax*

The target enzyme was partially purified from the liver, which was dissected from different individuals of *D*. *labrax* species. Tissue samples were homogenized in four volumes of ice-cold homogenization buffer (10 mM Tris/HCl, pH 7.5, containing 150 mM NaCl) with an Ultra-Turrax T25 homogenizer (IKA Works, Staufen, Germany). The homogenate was centrifuged at 288000 *g* for 1.5 h at 4°C. The supernatant was dialyzed against 25 mM Tris/HCl and 1 M ammonium sulfate (pH 7.5) (buffer A), and then applied to a Phenyl Sepharose column (1.6 x 2.5 cm) as described above. The eluted active fractions were pooled and stored in 25 mM Tris/HCl (pH 7.5) containing 5% glycerol.

The *D*. *labrax* hemolysate was prepared as described for *C*. *hamatus* and then loaded onto a DEAE Sepharose Fast-Flow column connected to an AKTA FPLC system and equilibrated in 25 mM Tris/HCl, pH 7.5 (buffer A). A linear gradient (0–100%) of 1 M NaCl in buffer A was used to elute the protein at a flow rate of 1 ml min^-1^. The active fractions were pooled, dialyzed against 25 mM Tris/HCl pH 7.5 and 1 M ammonium sulfate (buffer A) and then loaded on a Phenyl Sepharose column (1.6 x 2.5 cm) connected to an AKTA FPLC system, equilibrated with the same buffer. Bound proteins were eluted with a linear gradient (0–100%) of 25 mM Tris/HCl (pH 7.5) (buffer B) at a flow rate of 1 ml min^-1^. The APEH-1_*Dl*_ active fractions recovered were pooled, dialyzed against 25 mM Tris/HCl, pH 7.5 and loaded onto a Superdex 200 PC 3.2/30 column connected to a SMART System. The buffer of elution was 25 mM Tris/HCl, pH 7.5 and 50 mM NaCl and the flow rate was 0.1 ml min^-1^. Active fractions were pooled and the purified APEH-1_*Dl*_ was stored in 25 mM Tris/HCl pH 7.5 and 5% glycerol.

### Molecular mass determination

Protein homogeneity was estimated by SDS-PAGE analysis [29] with 8% (w/v) acrylamide resolving gel. Standard proteins (Broad Range) were from New England BioLabs. Molecular masses of the native enzymes were established by gel filtration chromatography on both Superdex 200 PC 3.2/30 or Superose 12 3.2/30 columns (Pharmacia Biotech), connected to a SMART System and calibrated with BioRad gel filtration standards (code 151–1901), as described in detail previously [12]. The protein concentration was determined with the Bradford assay method [30].

### Enzyme assays

The aminopeptidase activities of APEHs from *C*. *hamatus* and *D*. *labrax* were measured by the use of Ac-Leu-pNA (Acetyl-Leucine-para-nitroanilide, Sigma), Ac-Ala-pNA (Acetyl-Alanine-para-nitroanilide, Bachem), Ac-Phe-pNA (Acetyl-Phenylalanine-para-nitroanilide; Sigma) and Ac-Met-AMC (Bachem) as previously described [12].

### Temperature and pH influence on APEH activity

Determinations of temperature and pH optima of APEHs, as described in detail previously [12], were performed with Ac-Met-AMC as the substrate. The effect of pH was determined between pH 5.0 and pH 9.0 for *C*. *hamatus* APEHs and between pH 5.0 and pH 10.0 for *D*. *labrax* APEH, under the assay conditions described above. Citrate/phosphate buffer (50 mM) was used for pH 5.0, was replaced by Tris/HCl (50 mM) for pH 6.7–8.9 and by CAPS (50mM) for pH 9.5–10.0. Relative activity was expressed as percentage of the maximum of the enzyme activity under the standard assay conditions. Optimum temperatures for the enzymes were determined under the standard assay conditions in the temperature range 15–60°C for APEH-1_*Ch*_ and APEH-1_*Dl*_ and 15–50°C for APEH-2_*Ch*_. The thermal stabilities were determined by measuring residual activities on Ac-Met-AMC substrate after incubation of the enzymes at various temperatures (10–50°C). Temperatures below 10°C were not

Aliquots of protein samples, obtained explored, owing to their dramatic effects on substrate solubility.

### Western blot analysis

from blood (APEH-1_*Ch*_, APEH-2_*Ch*_ and APEH-1_*Dl*_) and liver (APEH-1_*Dl*_), were subjected to SDS-PAGE (8%), and then electroblotted onto polyvinylidene difluoride (PVDF) membranes (Immobilon; Millipore). Membranes were next incubated with the primary antibody (APEH N-18 goat IgG; 1: 5000; Santa Cruz Biotechnology) and then with the horseradish peroxidase-conjugated secondary antibody (1: 5000; 1 h at 37°C; Santa Cruz Biotechnology), as described in detail previously [12]. The immune complexes were visualized by enhanced chemiluminescence and autoradiography, according to the manufacturer’s protocol (Amersham Biosciences).

### N-terminal amino acid sequence analysis

Automated N-terminal sequence analysis of the two *C*. *hamatus* APEH isoforms (corresponding to 80 kDa and 75 kDa immunoreactive bands) electroblotted onto PVDF membranes (Bio-Rad) was performed with a Perkin-Elmer Applied Biosystems 477A pulsed-liquid protein sequencer. The PSI-BLAST program was used to scan the NCBI non redundant protein dataset [31], with the experimentally obtained N-terminus as the query sequence.

### Degradation of oxidized BSA by APEHs

A solution of unoxidized or oxidized BSA (1.7 μg), prepared as described by Fujino *et al*. 1998 [32], was treated with the APEHs (0.7 μg) from *C*. *hamatus* and *D*. *labrax* in 30 μl of 25 mM Tris/HCl (pH 8.0) at 37°C for 0, 24 and 48 h. 30 μl of the reaction mixtures were subjected to SDS-PAGE analysis. To compare the endopeptidase with the exopeptidase activity we expressed the oxidase protein endohydrolase activity (OPEH) in arbitrary units. One unit is defined as the amount of enzyme which hydrolyzes 1 μg of oxidized BSA at 37°C and 48 h.

## Results and Discussion

### Cloning of *C*. *hamatus* and *D*. *labrax apeh-1* and *apeh-2* cDNAs and phylogenetic analysis

To better understand the role of APEH in the antioxidant defence systems and in cold adaptation, we decided to characterise the two *C*. *hamatus* APEH isoforms by comparing their properties with those of a temperate counterpart. For this aim, the perciform sea bass *D*. *labrax*, which is one of the most commercially important seawater fish species in the Mediterranean Basin and is extensively bred in fish factories, was chosen as the piscine temperate model.

The partial cloning of the *apeh-1* and *apeh-2* cDNAs of *C*. *hamatus* and *D*. *labrax* was achieved with RT-PCR starting from total RNA extract from liver. The oligonucleotides utilised for the amplifications were designed based on the homologues sequences from other teleosts. The cloning of the *C*. *hamatus* cDNAs were facilitated by the knowledge of the homologous sequences from *T*. *bernacchii* because these two fishes belong to the same phylogenetic group, whereas the design of the primer pairs for the cloning of *D*. *labrax* partial cDNAs was obtained from the best conserved regions found in the alignment of the homologues sequences of teleosts retrieved from data banks. The isolated *C*. *hamatus apeh-1*_*Ch*_ and *apeh-2*_*Ch*_ partial cDNAs both covered almost the full coding region, corresponding to translations of 721 aa (APEH-1_*Ch*_) and 683 aa (APEH-2_*Ch*_) (S1 Fig). In contrast, the partial cDNAs of *D*. *labrax* both corresponded to a portion of the coding region 3’ end, with translations of 208 aa (APEH-1_*Dl*_) and 174 aa (APEH-2_*Dl*_) (S2 Fig).

**Fig 1:**
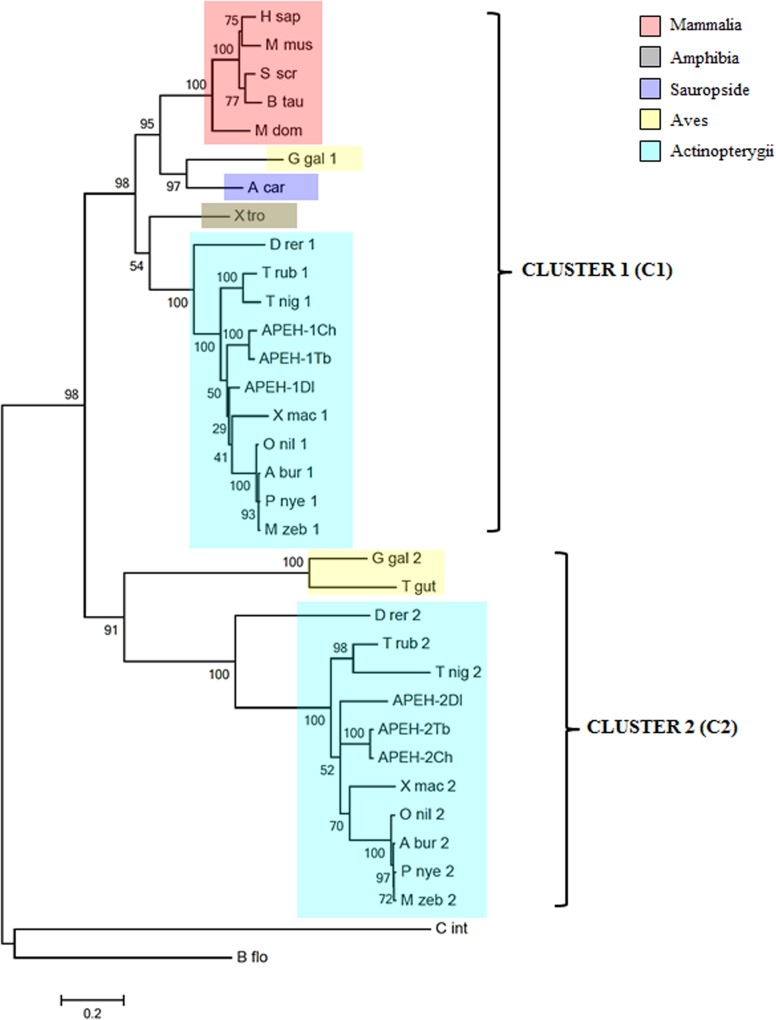
Phylogenetic analysis of APEHs proteins in the vertebrata *subphylum*. The cladogram includes the sequences retrieved from 22 organisms, two of which are invertebrates and were used as sister groups in the analysis. Numbers at nodes represent the confidence limits computed by the bootstrap procedure (100 replicates). All APEH sequences were found distributed into two distinct clusters: cluster 1 (C1) and cluster 2 (C2).

A comparative analysis between the deduced aa sequences of the four partial cDNAs and their homologues from some vertebrates and two invertebrates, which were retrieved from the Swiss-Prot/TrEMBL database (S1 Table and S3 Fig), was performed,. A single APEH chain was found for all the organisms analysed, with the exception of the teleosts and of a bird (*Gallus gallus*), where two APEH sequences were retrieved, APEH-1 and APEH-2. The APEH of two invertebrates (*Branchiostoma floridae* and *Ciona intestinalis*) were used as outgroups. A phylogenetic tree was constructed (Fig 1), sustained by high bootstrap in ML analysis. The analysis confirmed the finding of two clusters: cluster 1 (C1), containing all the single chains and the APEH-1 from the teleosts and the bird *Gallus gallus*; and cluster 2 (C2), that includes the APEH-2 isoform from the teleosts and from the birds *Gallus gallus* and *Taeniopygia guttata*. As expected, the APEH-1 and APEH-2 sequences from *C*. *hamatus* grouped with those from *T*. *bernacchii*, forming the respective nototheniod subclusters. Moreover, among all the other teleost sequences, those from *D*. *labrax* occupy the closest phylogenetic position with respect to the two Antarctic APEH-1 and APEH-2 subgroups, confirming the choice of this temperate fish as a very appropriate model to study the consequences of cold-adaptation for this family of proteins.

### Isolation and purification of APEHs from *D*. *labrax* and *C*. *hamatus*

**Fig 2:**
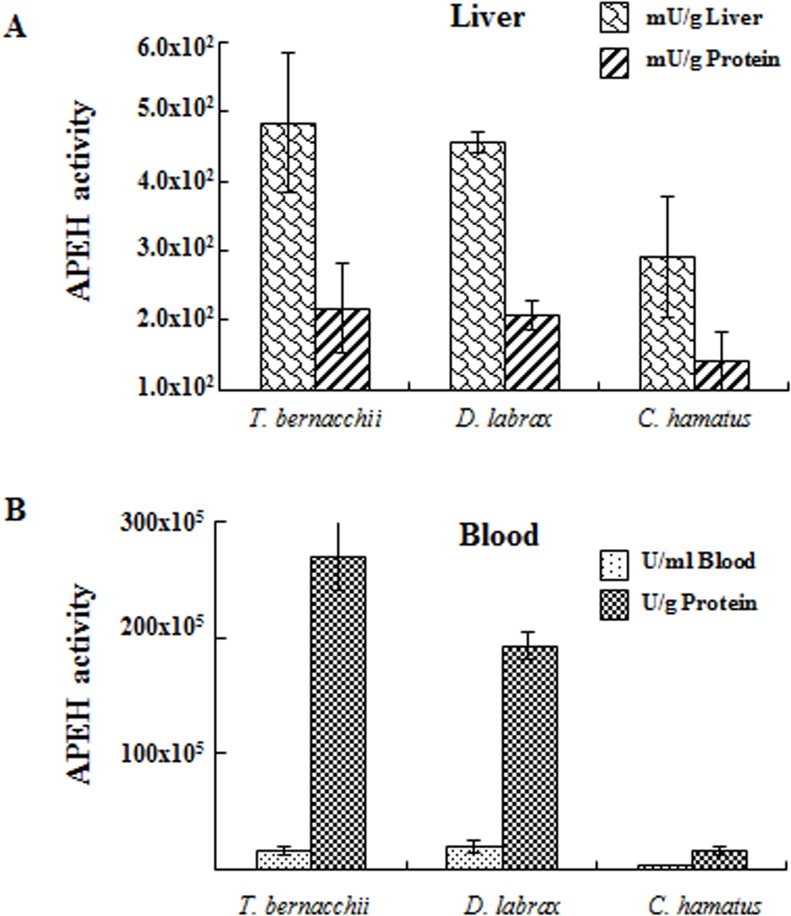
Total exopeptidase activity of APEH measured in (A) liver and (B) blood of *T*. *bernacchii*, *D*. *labrax* and *C*. *hamatus*. All the enzymatic activities were measured at 37°C using Ac-Met-AMC as substrate and expressed in arbitrary units. The results shown are representative of three independent experiments.

As a preliminary study, the total APEH exopeptidase activity was measured in protein extracts from blood and liver tissue from both *C*. *hamatus* and *D*. *labrax* species and compared to those of the red blooded Antarctic teleost, *Trematomus bernacchii*. Interestingly, as shown in Fig 2, although the APEH activity did not show substantial differences between *T*. *bernacchii* and *D*. *labrax* in both compartments, significantly lower values were observed in the protein extracts from *C*. *hamatus*, specifically in the blood possibly due to the extreme low viscosity of the haemoglobin-less cells compared to red-blooded counterparts.

#### a) *D*. *labrax*

**Fig 3:**
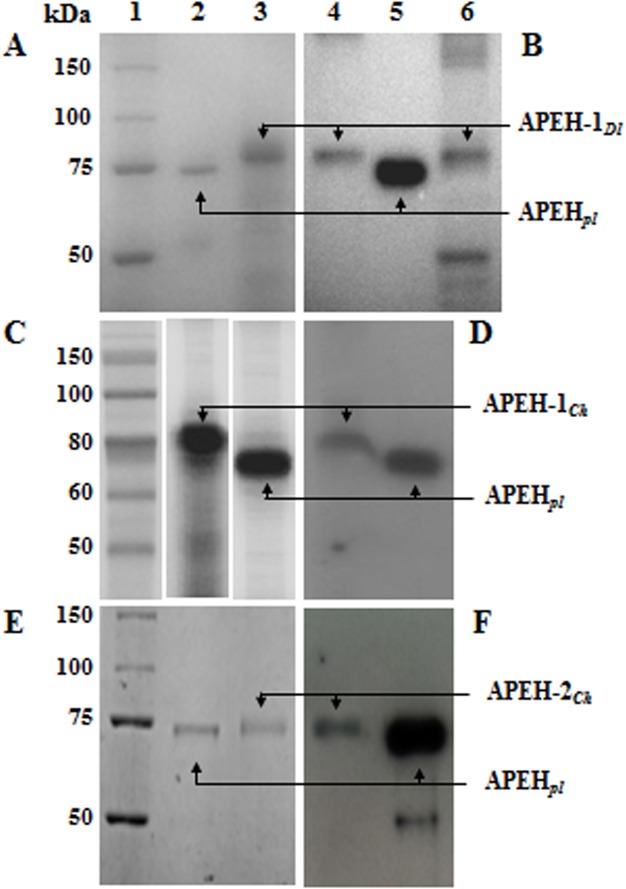
SDS-PAGE and immunoblotting analyses of APEH isoforms from the Antarctic and temperate fishes *C*. *hamatus* and *D*. *labrax*. (**A**) SDS-PAGE analysis of APEH-1_*Dl*_ purified from blood. Lane 1, Broad range (6.5–200 kDa) molecular weight markers (MWM) (Sigma-Aldrich); lane 2, APEH_*pl*_ from porcine liver used as control; lane 3, APEH-1_*Dl*_ purified from blood; (**B**) Immunoblotting analysis of APEH-1_*Dl*_. Lane 4, purified APEH-1_*Dl*_ from blood; lane 5, APEH_*pl*_; lane 6, APEH-1_*Dl*_ partially purified from liver. (**C**) SDS-PAGE analysis of APEH-1_*Ch*_. Lane 1, MWM Color Plus Prestained (BioLabs); lane 2, APEH-1_*Ch*_ purified from blood; lane 3, APEH_*pl*_. (**D**) Immunoblotting analysis of APEH-1_*Ch*_. Lane 4, APEH-1_*Ch*_ purified from blood; lane 5, APEH_*pl*_. (**E**) SDS-PAGE analysis of APEH-2_*Ch*_. Lane 1, MWM as well as lane 1 in A; lane 2, APEH_*pl*_; lane 3, APEH-2_*Ch*_ purified from blood. (**F**) Immunoblotting analysis of APEH-2_*Ch*_. Lane 4, APEH-2_*Ch*_ purified from blood; lane 5, APEH_*pl*_. The results shown are representative of three independent experiments.

The red blood cells (RBCs) were chosen as the starting tissue to optimise a purification procedure, which allowed the isolation of a unique APEH enzyme as no further isoform was identified in these cells. The enzyme was purified to homogeneity approximately 59-fold with an activity recovery of 17%, and a specific activity of 8.36 x 10^5^ U/mg in almost 0.90 mg (S2A Table). SDS-PAGE (Fig 3A) and Western blot (Fig 3B) analyses revealed an 80 kDa-band, and the enzyme exhibited an N-terminal ^1^AAESQPATEE sequence approximately matching the N-terminus (S3 Fig) of the members of the C1-APEH. Thus, this enzyme was named APEH-1_*Dl*_. To isolate the APEH-2 isoform, a purification procedure was set up starting from the liver, which is known as an APEH-rich tissue. As shown in Fig 3B, the unique immunodetected 80 kDa-band suggested the presence of the only APEH-1_*Dl*_, as also confirmed by Edman degradation analysis. Therefore, in a temperate species living in fish farms characterised by low stressor conditions, no enzyme activity was associated with APEH members belonging to the C2 group, which was believed to play a homeostatic role in sustaining the antioxidant defence systems.

#### b) *C*. *hamatus*

Based on a combination of hydrophobic affinity and ion exchange chromatography, a new APEH was isolated to homogeneity from the erythrocyte-like cells of the icefish *C*. *hamatus*. The protein was purified approximately 902-fold, with an activity recovery of 57% and a specific activity of 1.11 x 10^6^ U/mg as summarised in S2B Table. The homogeneity of the final product was determined by SDS-PAGE (Fig 3C), which revealed an 80 kDa-protein band that was also confirmed by Western-blot analysis (Fig 3D). The N-terminus was identified as ^2^GSQVVTN, which was consistent with that predicted from the coding gene *apeh-1*_*Ch*_, except for the first amino acid (S1A Fig), which remained uncertain even after repeated N-terminal sequence analysis. Therefore, this isoform was named APEH-1_*Ch*_.

Starting from the protein extract of the erythrocyte-like cells, the second APEH isoform was purified approximately 228-fold (S2B Table and Fig 3E) with a specific activity of 2.80 x 10^5^ U/mg. A unique 75 kDa-immunoreactive band (Fig 3F) and an N-terminus of MEPKQV, which matched the sequence predicted from the oligonucleotide previously designed for the *apeh-2*_*Ch*_ cDNA cloning, with the exception of a single position (S1B Fig), were identified. Then, this new isoform was named APEH-2_*Ch*_.

### Molecular properties of APEH isoforms from *C*. *hamatus* and *D*. *labrax*

Cold-active enzymes are a key determinant in life adaptation at low temperatures, but the adaptive optimisation of their catalytic parameters differs in amplitude and origin and the structural factors involved are diverse and complex [33].

The molecular properties of APEH-1_*Ch*_ and APEH-2_*Ch*_ were evaluated compared with those of the temperate APEH-1_*Dl*_. First, the degree of oligomerisation of the three purified APEHs was examined by gel filtration chromatography using two different size-exclusion columns. These analyses showed stable tetramers in solution for all APEH isoforms under investigation as also observed for the mammalian counterparts studied to date. The apparent molecular mass was approximately 300 kDa, composed of identical monomers of 80 kDa (APEH-1_*Ch*_ and APEH-1_*Dl*_) or 75 kDa (APEH-2_*Ch*_) (Fig 3). These values were consistent with those calculated on the basis of the amino acid sequences of *apeh-1*_*Ch*_ (80.9 kDa) and *apeh-2*_*Ch*_ (76 kDa) gene products (S1 Fig).

**Fig 4:**
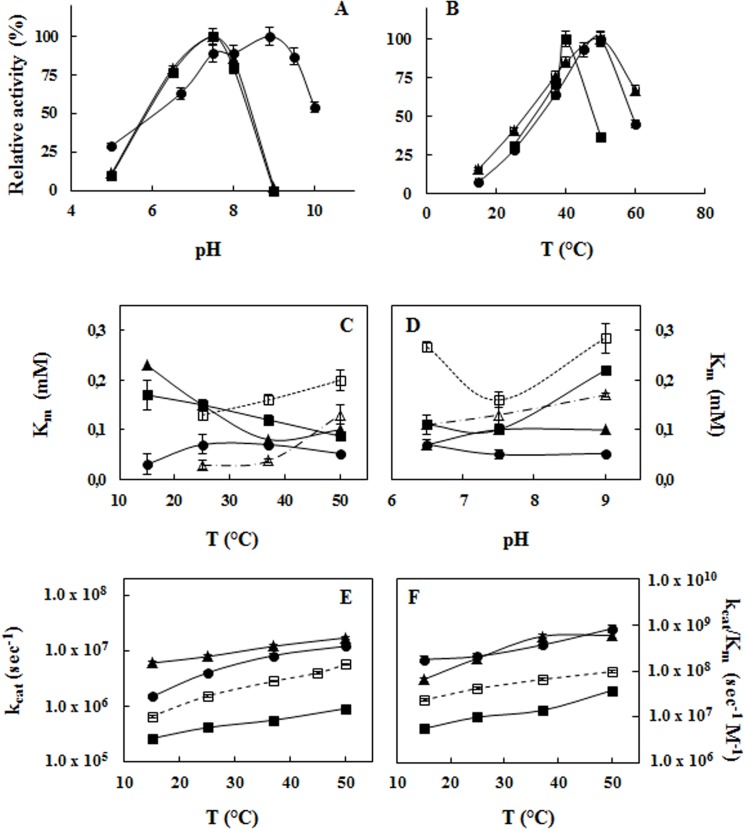
Effects of temperature and pH on catalytic parameters of *C*. *hamatus* APEH isoforms as compared with those of APEH-1_*Dl*_ from *D*. *labrax*. (**A**) pH–activity profiles of APEH-1_*Ch*_ (▲), APEH-2_*Ch*_ (■) and APEH-1_*Dl*_ (•). (**B**) Influence of temperature on enzyme activities. (**C**) Temperature–K_*m*_ profiles and (**D**) pH–K_*m*_ profiles of APEH-1_*Ch*_ (▲), APEH-2_*Ch*_ (■) and APEH-1_*Dl*_ (•) compared with those of APEH-1_*Tb*_ (△) and APEH-2_*Tb*_ (□) previously described [11] from *T*. *bernacchii*. Effect of temperature on (**E**) k_*cat*_ and (**F**) catalytic efficiency (k_*cat*_/K_*m*_) of analyzed APEHs. All experiments were performed in triplicate on three different protein preparations, using Ac-Met-AMC as substrate. Data are expressed as means ± standard deviations.

Furthermore, APEHs from *C*. *hamatus* showed identical bell-shaped pH-activity profiles with an optimal pH value of 7.5 (Fig 4A). Conversely, the response of APEH-1_*Dl*_ activity to pH was interesting in that two pH maxima at 7.5 and 8.9 seemed to be present. This pH phenomenon can result from the presence of different titratable groups close to the active site of APEH-1_*Dl*_, which affect the substrate or product binding and/or enzyme catalysis and therefore contribute to the shape and the alkaline shift in the pH activity profile.

Regarding the temperature-activity profiles, APEH-2_*Ch*_ displayed a sharp optimum at 40°C (Fig 4B) in contrast to that observed for APEH-1_*Ch*_ and APEH-1_*Dl*_, which both exhibited high activities over a broad temperature range (37–60°C) with an optimum at 50°C. Therefore, members belonging to cluster 2 (APEH-2_*Ch*_ and APEH-2_*Tb*_) [12], whose temperature for apparent maximal activity is shifted towards lower temperatures compared to those of cluster 1 (APEH-1_*Ch*_, APEH-1_*Tb*_, APEH-1_*Dl*_ and APEH_*pl*_) [12], are most likely cold-adapted enzymes. However, the optimal temperatures for the two *C*. *hamatus* APEH isoforms remained 25–30°C above the natural growth temperature for this Antarctic species (-1.9–2.0°C), as also observed for the APEHs from *T*. *bernacchii* and other psychrophilic enzymes [12,34,35]. This may suggest that the in vitro conditions do not reproduce physiological conditions in vivo of Antarctic fish and that several intracellular factors may greatly affect the stabilization of the protein and the enzyme-substrate recognition.

In addition, all of the piscine APEHs were highly stable at 10°C and showed a half-life of a few minutes at 50°C. However, the enzyme activity of APEH-1_*Ch*_ and APEH-2_*Ch*_ was already diminished by incubations at 20°C (residual activity was 60% and 80% after 5 h of incubation, respectively) whereas APEH-1_*Dl*_ appeared to be unaffected.

### Kinetic and thermodynamic parameters of APEH-1_*Ch*_, APEH-2_*Ch*_ and APEH-1_*Dl*_

Cold-adapted enzymes have been shown to use different structural adaptation strategies to increase molecular flexibility, such as weakening intramolecular hydrogen bonds, increasing the number of hydrophobic side chains exposed to the solvent, and reducing the number of salt bridges [36,37]. As a consequence of their high structural flexibility, most of the cold adapted enzymes usually increase k_cat_ (the turnover number) at the expense of K_m_ (substrate affinity) with a consequent loss of catalytic efficiency (k_cat_/K_m_) compared to the temperate-adapted homologs at the corresponding environmental temperatures [37]. Surprisingly, in contrast to the cold-adapted APEHs from *T*. *bernacchii*, both isoforms from *C*. *hamatus* showed better K_m_ values (using Ac-Met-AMC as substrate) at increasing temperatures, possibly due to the more hydrophobic nature of the β-propeller and/or α/β catalytic domains (Fig 4C). Actually, hydrophobic interactions, involved in substrate binding, are strengthened as the temperature increases at the expense of electrostatic interactions yielding high binding affinities. In addition, as observed for several enzymes from eurythermal marine species [37,38], the K_m_ values of APEH-1_*Dl*_ were relatively unaffected in the 15–50°C temperature range and were also the lowest measured at all temperatures (Fig 4C).

The different behaviour in terms of substrate affinity (K_m_) of the three APEHs is further highlighted based on the pH-kinetic response profiles (Fig 4D). Indeed, the overall variations in K_m_ with pH were not significantly altered for APEH-1_*Ch*_ and APEH-1_*Dl*_ in the pH range 6.5–9.0, as also observed for APEH-1_*Tb*_. On the other hand, the pH-K_m_ profile of APEH-2_*Ch*_, which is common to APEH-2_*Tb*_, assumed a convex shape with a minimum at pH 7.5, highlighting a specific kinetic pH window due to a change between the C1- and C2-APEH members in the electrostatic environment around their active sites.

The effect of catalytic properties on the biological function of enzymes can also be studied by correlating the substrate specificity with physiological variations. In this context, a comparison among the optimal K_m_ parameters based on four different acyl-amino acid substrates (Table 2) of the piscine APEHs revealed that both *C*. *hamatus* isoforms showed a broad specificity but lower substrate affinities in contrast to APEH-1_*Dl*_, which was more selective and efficient in recognition of Ac-Ala and Ac-Met.

**Table 2:** Optimal kinetics parameters of *C*. *hamatus* APEHs in comparison with those from *D*. *labrax*.

Substrate	Enzyme	K_m_	Temperature
Exopeptidase activity		(mM)	°C
**Ac-Ala-pNA**	**APEH-2**_***Ch***_	8.3x10^-2^	37
	**APEH-1**_***Ch***_	10 x10^-2^	15
	**APEH-1**_***Dl***_	2.8 x10^-2^	15
**Ac-Met-AMC**	**APEH-2**_***Ch***_	30 x10^-2^	15
	**APEH-1**_***Ch***_	21 x10^-2^	15
	**APEH-1**_***Dl***_	5.2 x10^-2^	15
**Ac-Leu-pNA**	**APEH-2**_***Ch***_	17 x10^-2^	37
	**APEH-1**_***Ch***_	10 x10^-2^	25
	**APEH-1**_***Dl***_	n.a.	-
**Ac-Phe-pNA**	**APEH-2**_***Ch***_	22 x10^-2^	50
	**APEH-1**_***Ch***_	n.a.	-
	**APEH-1**_***Dl***_	n.a.	-

Kinetic parameters measured using Ac-Met-AMC as substrate are expressed in arbitrary units.

Regarding the kinetic constants, APEH-1_*Ch*_ and APEH-1_*Dl*_ exhibited better k_cat_ at increasing temperatures, although the values determined for APEH-1_*Ch*_ were slightly higher than those of the temperate-acclimated counterpart in the temperature range analysed (Fig 4E). Surprisingly, very similar temperature-(k_cat_/K_m_) profiles were observed between the piscine (Fig 4F) and mammalian [12] members of C1-APEH, despite the significant differences in their environmental conditions. Conversely, the k_cat_ and catalytic efficiency (k_cat_/K_m_) values relative to APEH-2_*Ch*_ were approximately two orders of magnitude lower at all temperatures tested (Fig 4E and 4F).

The enzymes of cold-adapted species typically operate at higher k_cat_ values specifically at their physiological temperatures, than the orthologous from temperate organisms [37]. This behaviour was shown by APEH-1_*Ch*_, whose temperature compensation in performance capacity appeared to be complete. On the contrary, the APEH-2 isoforms from the Antarctic fish represent one of the several exceptions to this general rule. We hypothesize that in the icefish blood, APEH isoforms are both expressed to compensate the low k_cat_/K_m_ values of APEH-2_*Ch*_ although the total APEH activity in this tissue (as shown in Fig 2) remains lower than that measured in *T*. *bernacchi* and *D*. *labrax*, possibly due to a minor degree of protein turnover related to their lower metabolic rate.

Hence, the APEHs can adopt diverse strategies to allow a fine kinetic optimisation by modifying the thermodynamic and kinetic properties of the weak interactions that are involved in the catalytic performances.

### Oxidised protein endohydrolase (OPEH) activity of APEH isoforms from *C*. *hamatus*

Although the exact biological role of APEH has not yet been completely defined, three functions can be described: (1) exopeptidase activity that unblocks N-acetyl peptides [13,39]; (2) endopeptidase activity towards protein aggregates induced in erythrocytes membrane by oxidative stress [32,40,41] and (3) ability to associate with aggresomes when proteasome function is down regulated [42]. In addition, we suggested that APEH acts in coordination with proteasomes in the protein degradation machinery, suggesting a role in the modulation of the ubiquitin-proteasome pathway and *clearance* of oxidised proteins [43]. Recently, we also identified a new cluster of APEH from *T*. *bernacchii*, which can participate in ROS detoxification as a phase 3 antioxidant enzyme [12].

**Fig 5:**
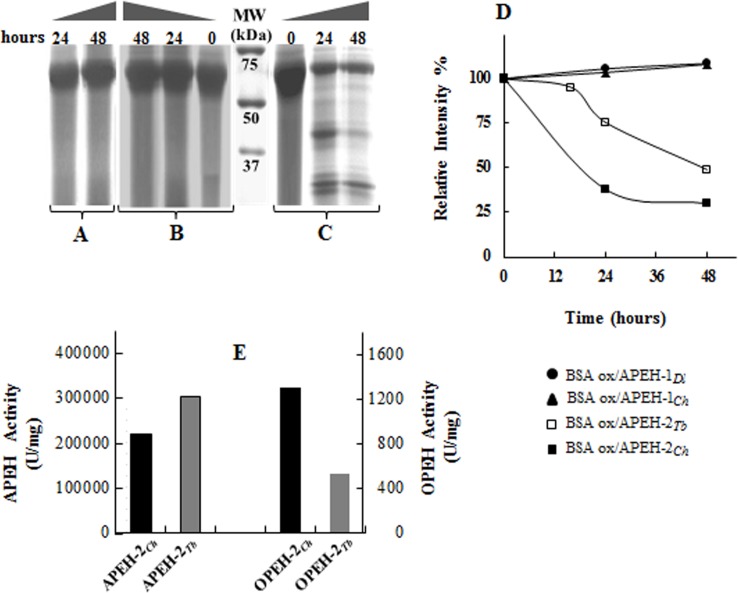
Oxidised protein endohydrolase (OPEH) activity using BSA as substrate. (**A**) SDS-PAGE analysis of oxidised BSA incubated at 37°C without enzyme used as control. (**B**) Representative SDS-PAGE profile of oxidised BSA treated with APEH-1_*Ch*_ and APEH-1_*Dl*_ at 37°C. The same results were obtained after incubation of unoxidised BSA with all APEH isoforms (data not shown). (**C**) SDS-PAGE analysis of oxidised BSA treated at 37°C with APEH-2_*Ch*_. (**D**) Electrophoretic data expressed as percentage density of BSA at the indicated incubation times versus time 0 obtained by densitometric analysis with CHEMIDOC XRS and QUANTITY ONE software. Oxidised BSA levels after incubation with APEH-2_*Tb*_ were reported as comparative data [12]. (**E**) Exopeptidase (APEH) *vs* endoprotease (OPEH) activity of APEH-2_*Ch*_ and APEH-2_*Tb*_. All the experiments were performed in duplicate on two different protein preparations, and the average of the relative intensities of measurements, performed in triplicate are expressed as means ± standard deviation (values lower than 5% are not shown).

To better clarify the physiological role of APEH under environmental oxidative stress conditions, we examined the oxidised protein hydrolase activity of APEH-1 and APEH-2 from *C*. *hamatus* compared with that of the cognate APEH isoforms from *T*. *bernacchii*, using BSA as a substrate model and APEH-1_*Dl*_ as temperate control. Reaction mixtures containing unoxidised or oxidised BSA incubated with or without APEHs, were subjected to SDS-PAGE analysis. As shown in Fig 5C and 5D, a remarkable decrease of the intensity of the oxidised BSA band was observed following treatment with APEH-2_*Ch*_ with the detection of lower molecular mass fragments at 24 h, which were further hydrolysed until 48 h of incubation. Interestingly, this antioxidant proteolytic activity appeared to be much more efficient than that measured for APEH-2_*Tb*_ (Fig 5D), as also shown in Fig 5E, in which a comparison between exopeptidase (APEH) and endoprotease (OPEH) activities (expressed in arbitrary units) of APEH-2_*Ch*_ and APEH-2_*Tb*_ is shown. Oxidised BSA incubated without APEHs was used as control (Fig 5A). Conversely, no substrate reduction was detected following APEH-1_*Ch*_ (Fig 5B) or APEH-1_*Dl*_ (data not shown) incubations, as also observed for APEH-1_*Tb*_ [12]. In addition, no BSA fragments were revealed when unoxidised BSA was used as a substrate with all the APEHs under investigation, confirming the ‘atypical’ endoprotease activity associated with the eukaryal members of C2-APEH. Indeed, it has been suggested that in the oxidised BSA occurs a conformational change that brings the cleavage sites to the surface of the molecule, making them susceptible to degradation by APEH [41].

### Molecular modelling and structural analysis

**Fig 6:**
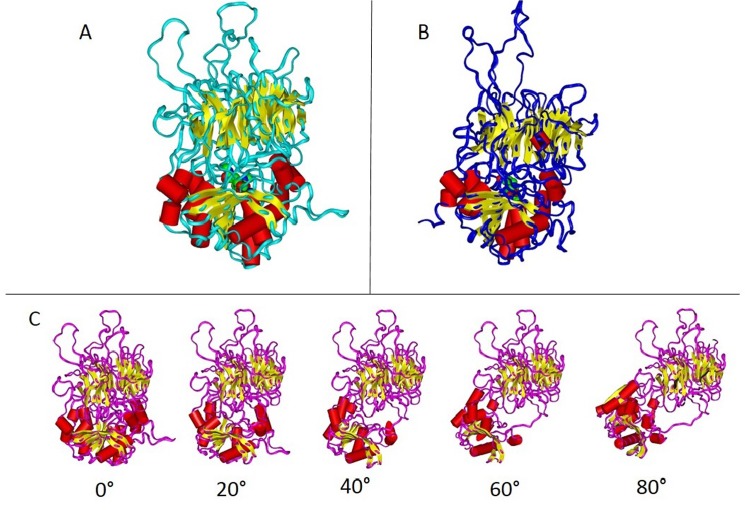
3D models of APEHs from *C*. *hamatus*. The structural architecture of APEH-1_*Ch*_ (panel **A**) and APEH-2_*Ch*_ (panel **B**) shows an N-terminal domains characterized by β strands (yellow arrows) while the C-terminal domains are characterized by β strands and α helices (red cylinders). Note that some non-structured segments on the top of the figure are segments modelled without reference in the template, due to gaps in the alignment. In panel **C**, we show the effects of rotations of 20°, 40°, 60° and 80° around a backbone bond of APEH-2_*Ch*_ along the segment 451–460, which connects the two domains. In this specific example, the rotation has been performed at the N-Cα bond of 454 residue. Similar effects were observed by rotating other backbone bonds (both N-Cα or Cα-C) at different positions of the 451–460 segment.

Homology modelling has been applied to model the 3D structure of APEH-1_*Ch*_ and APEH-2_*Ch*_ in consideration of the availability of the 3D structure of the APEH from *Aeropyrum pernix*, which shares approximately 20% sequence identity and 30% sequence similarity with both APEH-1_*Ch*_ and APEH-2_*Ch*_. The detailed modelling procedure (see Materials and Methods) includes the creation of more models for each protein and the selection of the best model based on a quality evaluation. The best model selected for both proteins has more than 95% of its residues in the core and allowed regions of the Ramachandran plot (not shown). The structural models of APEH-1_*Ch*_ and APEH-2_*Ch*_ are shown in Fig 6A and 6B. As expected, APEH-1_*Ch*_ and APEH-2_*Ch*_ models show the typical architectural organisation of all known APEHs with two domains separated by a segment of ten amino acids. The N-terminal domain displays a β-propeller structure, whereas the C-terminal region is organised as an α/β domain. No apparent or significant differences between the two proteins are evidenced by the 3D models. However, similarly to that observed for *T*. *bernacchii* APEHs [12], the two forms from *C*. *hamatus* display a different composition of the catalytic triad environment, with a predominance of negative charges in APEH-1 isoform and positive charges in APEH-2 isoform (S3 Table).

Moreover, by considering that the connecting segments between the two domains could act as a hinge that opens or closes access to the catalytic site, we simulated the rotation around a single backbone bond within the segment, and observed that the structure exposes this site making the substrate access easier (Fig 6C). The same effect was obtained by rotations around different bonds along with the connecting region (not shown). This segment is not folded as a specific secondary structure element, and its flexibility may contribute to the accessibility to the substrate, although further details are required to elucidate the underlying mechanism of this modulation, making some APEHs more active than others. Specifically, inter-domain interactions are important determinants of the protein adaptation to substrates and to environmental conditions.

Therefore, to gain insight into the structural features that can help understand the biological functions associated with the two enzyme isoforms, we investigated the details of the inter-domain bond formation by salt bridges and H-bonds as well as the charge distributions within the proteins. The results of this investigation are reported in Table 3, which includes all of the data obtained in parallel with *T*. *bernacchii* APEH enzymes to compare and highlight the specific structural features of the proteins from the two Antarctic organisms. This analysis clearly indicated that both APEH-2 isoforms are characterised by a high positive net charge compared to APEH-1 enzymes showing a prevalence of negative charges, which can be responsible for a different substrate binding mechanism. This different charge distribution explains the ability of APEH-2 isoforms to interact with the negatively charged methionine sulfoxide, a common modification of oxidised proteins. Moreover, in *T*. *bernacchii* the two isoforms have the same number of salt bridges whereas there are fewer H-bond interactions in APEH-1_*Tb*_ compared to APEH-2_*Tb*_, suggesting that the latter has a more rigid structure. The opposite effect is shown for *C*. *hamatus* proteins; APEH-1_*Ch*_ contains a higher number of H-bonds and salt bridges, indicating that APEH-2_*Ch*_ may be more flexible than APEH-1_*Ch*_. In addition, an in-depth analysis has been focused on the capability of the two domains to attract or repulse each other, giving an easier access to the catalytic site by a structural transition, from closed to opened conformation. This ability is the result of a complex mechanism that includes the stability and the structural flexibility of the protein, but it is difficult to understand how the different forces balance each other and determine the structural transition. In the inter-domain interactions, the relative numbers of salt bridges or H-bonds are as follows: APEH-1_*Tb*_ = APEH-2_*Tb*_<APEH-1_*Ch*_<APEH-2_*Ch*_ or APEH-1_*Tb*_<APEH-2_*Ch*_ <APEH-1_*Ch*_<APEH-2_*Tb*_, which does not reflect the membership of isoforms to the two clusters (C1 or C2) and could not be directly correlated with the variations in the enzymatic functions. Moreover, the net charge of domains can be taken into consideration to evaluate the mutual mobility of the two domains, as well as the recognition mechanism towards the different substrates. Specifically, APEH-1_*Tb*_ has both domains with a negative net charge, which may generate a repulsive effect making easier the exposure of the active site to the substrate. On the contrary, the two domains in APEH-1_*Ch*_ have opposite net charge, i.e., +1 and -2, suggesting an electrostatic attractive force between them, which can make opening of the structure and the access of the substrate to the catalytic site more difficult, compared to APEH-1_*Tb*_. The APEH-2s have a high positive net charge in both domains, strongly favouring the opening transition. Therefore, based on the net charge of the two domains, the relative propensity to open the structure is as follows: APEH-1_*Ch*_<<APEH-1_*Tb*_ <APEH-2_*Tb*_<APEH-2_*Ch*_.

**Table 3:** Charges distribution, inter-domain salt bridges and H-bonds in the 3D models of APEHs from *T*. *bernacchii* and *C*. *hamatus*.

	*T*. *bernacchii*				*C*. *hamatus*			
	APEH-1		APEH-2		APEH-1		APEH-2	
	N-ter	C-ter	N-ter	C-ter	N-ter	C-ter	N-ter	C-ter
**Sequence region**	1–450	461–728	1–420	431–690	1–450	461–729	1–420	431–690
**Total negative charges (D+E)**	64	29	42	24	56	29	46	24
**Total positive charges(H+K+R)**	58	26	51	31	57	27	53	31
**Net charge**	-6	-3	9	7	1	-2	7	7
**Total Protein net charge**	-9		16		-1		14	
**Total Protein Salt bridges**	20		20		16		11	
**Inter-domain salt bridges**	2		2		4		5	
**Total Protein H-bonds**	445		465		482		456	
**Inter-domain H-bonds**	33		57		48		46	

Finally, while it is not possible to clarify which effect mainly contributes to the protein flexibility, some specific structural features, such as the total protein/domain net charge, are shared by APEH-2 members and could be responsible for their higher propensity towards opening of the structure. Therefore, these observations offer an explanation of the ability of APEH-2s to hydrolyse internal peptide bonds of oxidised proteins, in contrast to APEH-1s, allowing the catalytic site to interact with bulky substrates.

### Expression analysis of *apeh-1* and *apeh-2* genes in *C*. *hamatus* and *D*. *labrax*

The expression levels of the *apeh-1* and *apeh-2* genes were analysed in several cells and tissues of *C*. *hamatus* and *D*. *labrax* by qRT-PCR [28].

#### a) *C*. *hamatus*

**Fig 7:**
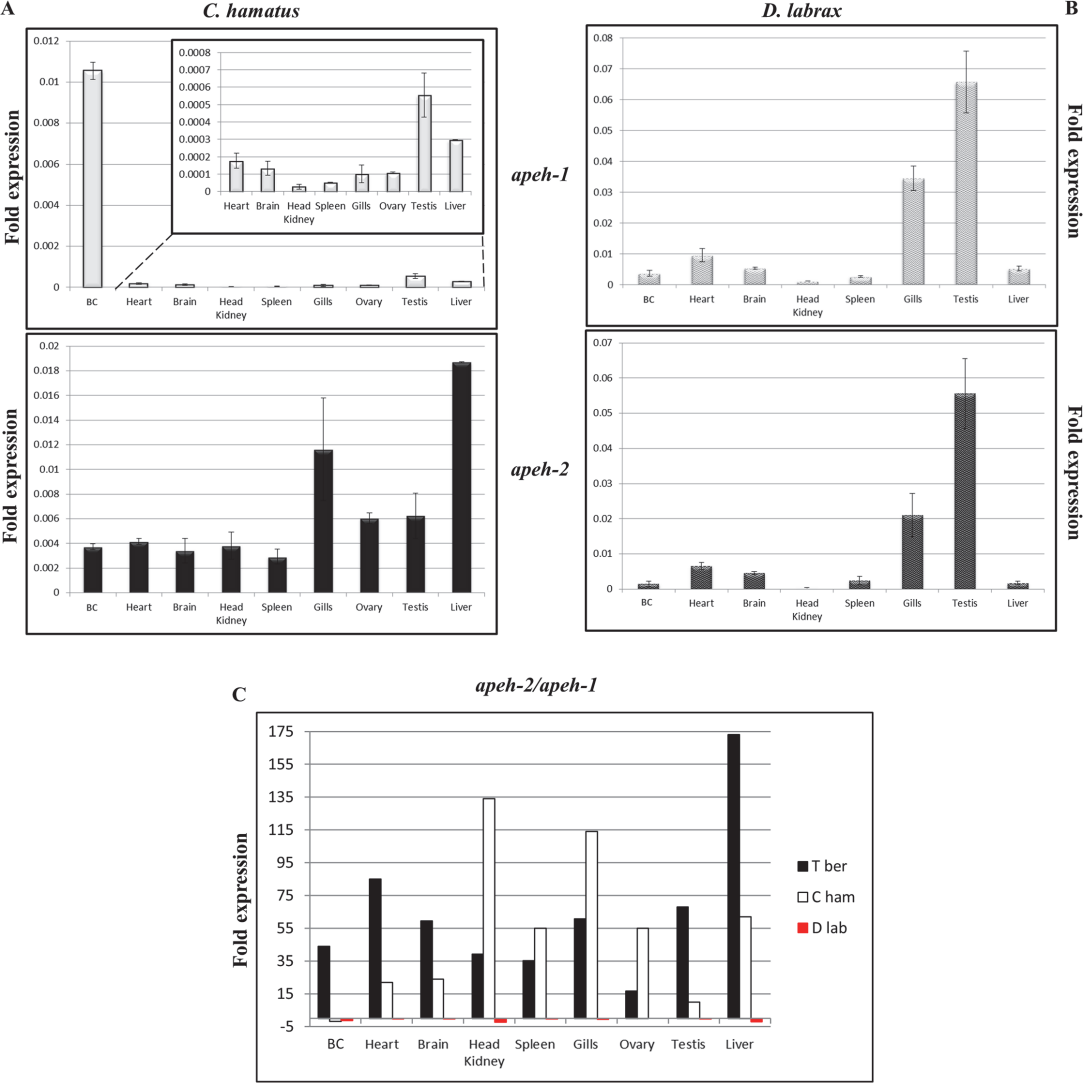
Expression analysis of *apeh* genes from *C*. *hamatus* and *D*. *labrax*. Analysis of the expression levels of *apeh-1* and *apeh-2* genes in cells and tissues of (**A**) *C*. *hamatus* and (**B**) *D*. *labrax*, normalised respect to the β-actin gene. The inset in the upper box of panel **A** contains a magnification for tissues with low expression levels. (**C**) The ratios of the expression folds obtained for the two *apeh* genes (*apeh-2*/*apeh-1*) in *C*. *hamatus* and *D*. *labrax* are shown compared to the relative values found in *T*. *bernacchii* [12]. All data are expressed as mean expression fold from triplicates.

The expression patterns obtained are shown in Fig 7A. The levels of *apeh-1*_*Ch*_ in the blood cells (BC) were the highest, with values ranging from 20 to 200 times those of all the other tissues analysed; among these other tissues, the highest levels were found in the testis followed by the liver. The *apeh-2*_*Ch*_ gene exhibited higher expression in the liver and gills, in that order, whereas in the other tissues, the mRNA levels were quite comparable. Moreover, by analysing the two gene expression profiles, *apeh-2*_*Ch*_ showed higher expression compared to *apeh-1*_*Ch*_ in all the tissues except for blood, with an *apeh-2*_*Ch*_ / *apeh-1*_*Ch*_ ratio ranging from 0.35 times in BC to 135 times in the kidney (Fig 7C).

#### b) *D*. *labrax*

Fig 7B shows the expression levels of the two *apeh* genes. In contrast to observations in *C*. *hamatus* and previously in *T*. *bernacchii* [12], *apeh-1*_*Dl*_ and *apeh-2*_*Dl*_ displayed very similar transcriptional patterns in terms of their tissue distribution and expression levels. In fact, both genes exhibited major transcriptional levels in the testis and gills, whereas in the other tissues, their expression was comparable. Thus, the fold-ratio of *apeh-2*_*Dl*_ / *apeh-1*_*Dl*_ was close to 1 in all tissues, although in some cases, a slight predominance of *apeh-1*_*Dl*_ transcripts was observed (Fig 7C). Altogether, by comparing the expression patterns of the *apeh* genes across different Antarctic or temperate piscine species, it was possible to highlight adaptive phenomena favoured by the different environmental conditions, although some divergences were observed possibly related to the presence/absence of haemoglobin in the two Antarctic fish.

Our data indicate that the Antarctic marine environment requires the presence of an enzymatic machinery supported by APEH-2 in specific tissues, to drive the degradation of oxidatively damaged proteins, whose amount becomes significant due to the high concentration of oxygen generated by the low temperatures. The differences in the transcriptional levels ratio *apeh-2* / *apeh-1* observed in the blood of the two Antarctic species, sustained by the measured levels of proteins, which identify a reduced demand for APEH-2 in *C*. *hamatus*, may be explained by taking into account the unique haemoglobin-less feature of the icefish family. Indeed, this condition makes their blood less prone to the accumulation of oxidised proteins due to the lack of haem, which under oxidative stress can act as a Fenton’s reagent to catalyse the production of free radicals in an unfettered manner [44,45]. In addition, the very high expression level of *apeh-1* transcripts in the blood of *C*. *hamatus* accompanied by the presence of the APEH-1 protein, which was not found in the red blood cells (RBCs) of *T*. *bernacchii*, could indicate that the icefish erythrocyte-like cells require the APEH-1 isoform to compensate the reduction of the contribution of APEH-2 as exopeptidase. Finally, this investigation revealed that the expression of the two *apeh* genes is post-transcriptionally regulated in all three fish: *i*) in the two Antarctic fish, where the almost always predominant *apeh-2* mRNA did not correspond to a major relative amount of the APEH-2 isoform, the expression levels were not proportional to the protein amounts; *ii*) in *D*. *labrax*, where *apeh-2* transcripts were detected in all the analysed tissues but the APEH-2 isoform was never noticed. Therefore, it is possible that specific microRNAs are involved in the modulation of gene expression through post-transcriptional regulatory mechanisms *via* the RNA-interference that is often associated with the response to cell stress conditions [46,47].

## Conclusions

Icefish are a unique example of adult vertebrates that, lacking haemoglobin, show an improvement in their antioxidant defences through a variety of mechanisms [48,49].

In this study, we characterised a new piscine enzyme belonging to cluster 2 (C2) from the icefish *C*. *hamatus*, named APEH-2_*Ch*_, and confirmed the high efficiency of these members in hydrolysing oxidised proteins and their involvement in ROS detoxification as phase 3 enzymes [32,40–42]. Our results also revealed that C2-APEHs can be stress regulated proteins that are mostly distributed in erythrocyte cells (S4 Fig), playing a homeostatic role in enabling the life of Antarctic fish under cold and oxidative stress conditions. Conversely, the APEHs belonging to cluster 1 (C1) (i.e., APEH-1_*Ch*_, APEH-1_*Dl*_ and APEH-1_*Tb*_) could represent the constitutive and most conserved isoforms in terms of molecular functions reflecting common structural determinants across eukaryal species.

Therefore, a comparative modelling analysis of *T*. *bernacchii* and *C*. *hamatus* APEHs, provided the basis for future investigations of the biochemical characterisation of this enigmatic enzyme family. Although it is not possible to evaluate the complete balance of the contribution to the architectural stability, a simple simulation of the opening of the protein structures indicates the mechanism of substrate recruitment due to the flexibility of the hinge segment connecting the two domains, allowing the access of the substrate to the active site. We proposed that the balance of the electrostatic forces between the N- and C-terminal domains, together with the inter-domain salt bridges, the H-bonds and the net charge of the catalytic site environment, determine the different enzymatic behaviour of the two isoforms.

Considering the gene expression results, our analysis shows a different tissue specificity of the *apeh* gene, which seems to be related to the diverse role of the two isoforms and to the particular physiological needs of the organism.

## Supporting Information

S1 Fig Sequences of *apeh-1*_Ch_ (A) and *apeh-2*_Ch_ (B) cDNAs from *C*. *hamatus* with the deduced amino acid sequences. The amplification primers are shown (green arrows). The N-terminal amino acid sequence of the proteins APEH-1_*Ch*_ (A) and APEH-2_*Ch*_ (B) are shown in red.
(PDF)

S2 Fig Partial sequences of *apeh-1*_Dl_ (A) and *apeh-2*_Dl_ (B) cDNAs from *D*. *labrax* with the deduced amino acid sequences. The amplification primers are shown (green arrows).
(PDF)

S3 Fig MUSCLE alignment of APEH-1 and APEH-2 sequences from different sources. The consensus sequence, the conservation histogram and the sequence logo are shown at the bottom of the alignment.
(PDF)

S4 Fig Western blot analysis of APEH-1 and APEH-2 isoforms in blood cells and different tissues from *C*. *hamatus* and *D*. *labrax*. Purified APEH-1 and APEH-2 from blood cells of both fish and the commercially available APEH from porcine liver (APEH_*pl*_) were loaded as controls. All the experiments were performed in duplicate on two different protein preparations.
(PDF)

S1 Table APEH chains utilized for phylogenetic analysis. (PDF)

S2 Table Purification of APEH isoforms from *D*. *labrax* (A) and C. hamatus (B). (PDF)

S3 Table Catalytic site environment for APEH-1_*Ch*_ and APEH-2_*Ch*_. Side chain atoms within 3 Angstroms from the catalytic triade in APEH-1_*Ch*_ (i.e. Ser585, Asp673, His705) and APEH-2_*Ch*_ (i.e. Ser555, Asp643, His675) are listed. The column with notes indicate when amino acid is part of the catalytic triade, and when atoms from side chains are charged.
(PDF)
