# Supplementary material for: A New APEH Cluster with Antioxidant Functions in the Antarctic Hemoglobinless Icefish Chionodraco hamatus
Source: PLoS One. 2015 May 6;10(5):e0125594. doi: 10.1371/journal.pone.0125594 (PMC4422685; doi:10.1371/journal.pone.0125594)
Supplement: S1 Fig — The amplification primers are shown (green arrows). The N-terminal amino acid sequence of the proteins APEH-1Ch (A) and APEH-2Ch (B) are shown in red. (PDF) [file pone.0125594.s001.pdf]

C. hamatus APEH cDNAs

A

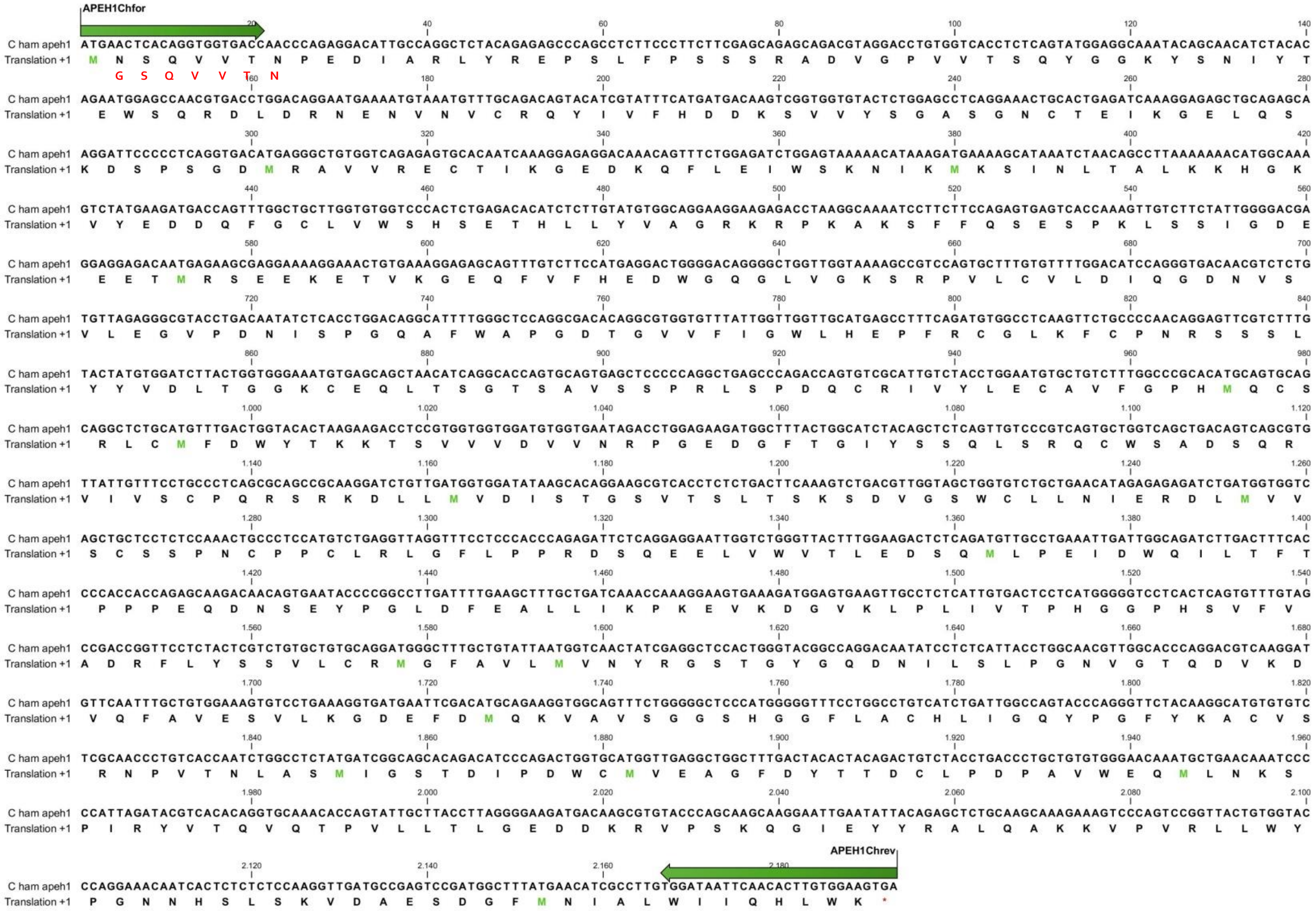

B

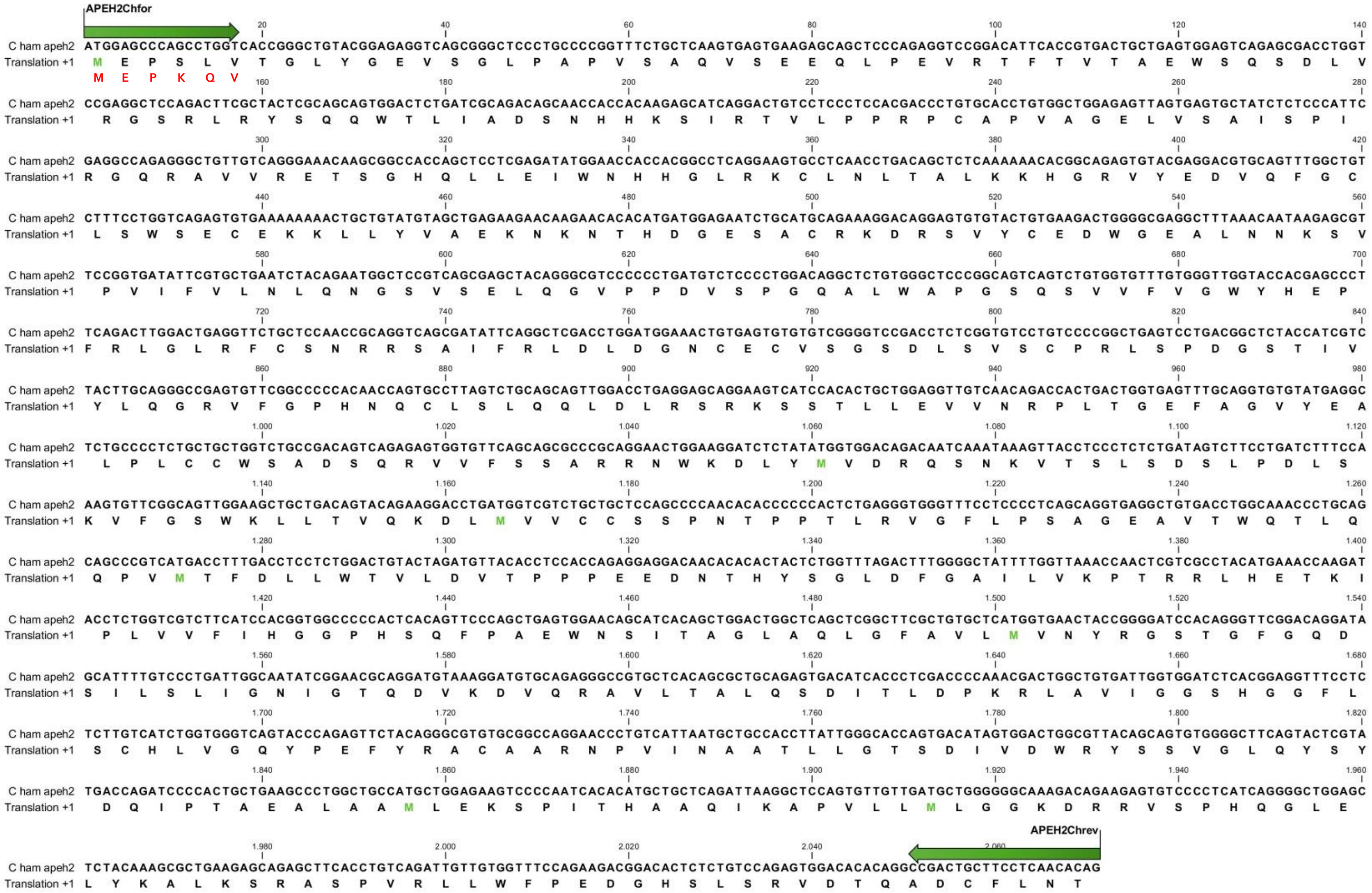

Figure S1. Sequences of *apeh*-1$_{Ch}$ (**A**) and *apeh*-2$_{Ch}$ (**B**) cDNAs from *C. hamatus* with the deduced amino acid sequences. The amplification primers are shown (green arrows). The N-terminal amino acid sequence of the proteins APEH-1$_{Ch}$ (**A**) and APEH-2$_{Ch}$ (**B**) are shown in red.
