# Supplementary material for: A New APEH Cluster with Antioxidant Functions in the Antarctic Hemoglobinless Icefish Chionodraco hamatus
Source: PLoS One. 2015 May 6;10(5):e0125594. doi: 10.1371/journal.pone.0125594 (PMC4422685; doi:10.1371/journal.pone.0125594)
Supplement: S2 Fig — The amplification primers are shown (green arrows). (PDF) [file pone.0125594.s002.pdf]

D. labrax APEH cDNA fragments

A

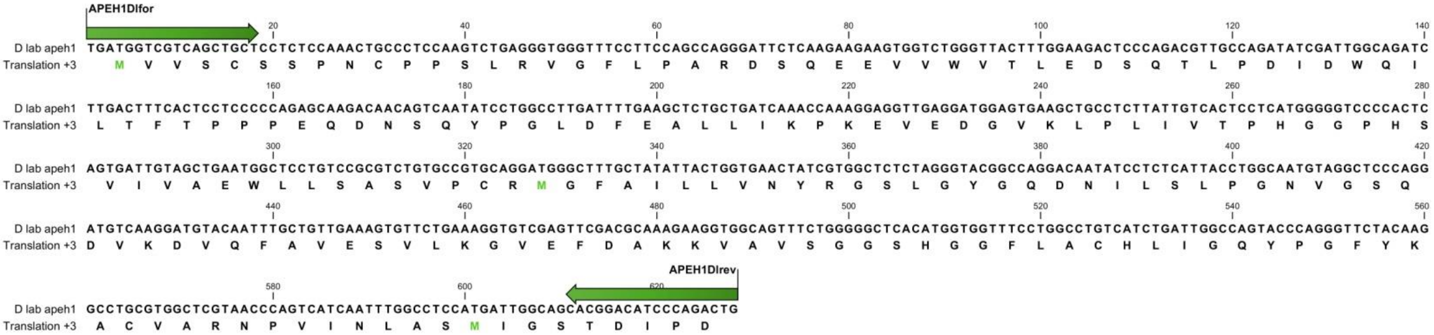

B

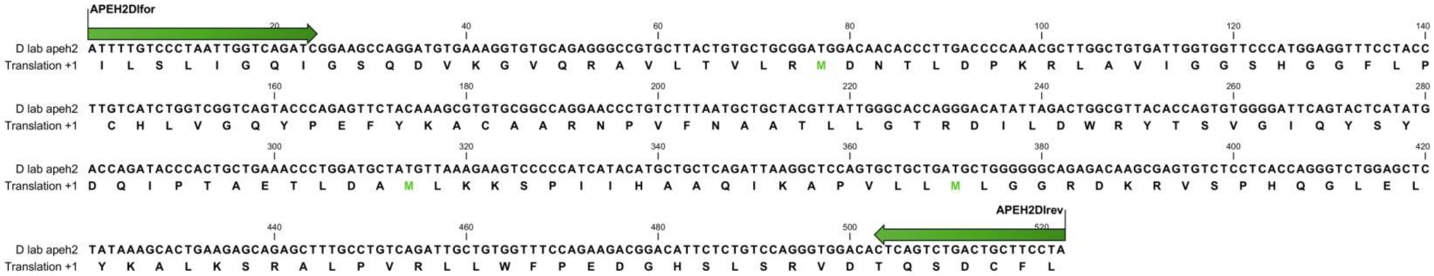

Figure S2. Partial sequences of *apeh-1*$_{Dl}$ (**A**) and *apeh*-2$_{Dl}$ (**B**) cDNAs from *D. labrax* with the deduced amino acid sequences. The amplification primers are shown (green arrows).
