# Supplementary material for: A New APEH Cluster with Antioxidant Functions in the Antarctic Hemoglobinless Icefish Chionodraco hamatus
Source: PLoS One. 2015 May 6;10(5):e0125594. doi: 10.1371/journal.pone.0125594 (PMC4422685; doi:10.1371/journal.pone.0125594)
Supplement: S3 Fig — The consensus sequence, the conservation histogram and the sequence logo are shown at the bottom of the alignment. (PDF) [file pone.0125594.s003.pdf]

APEH sequences alignment

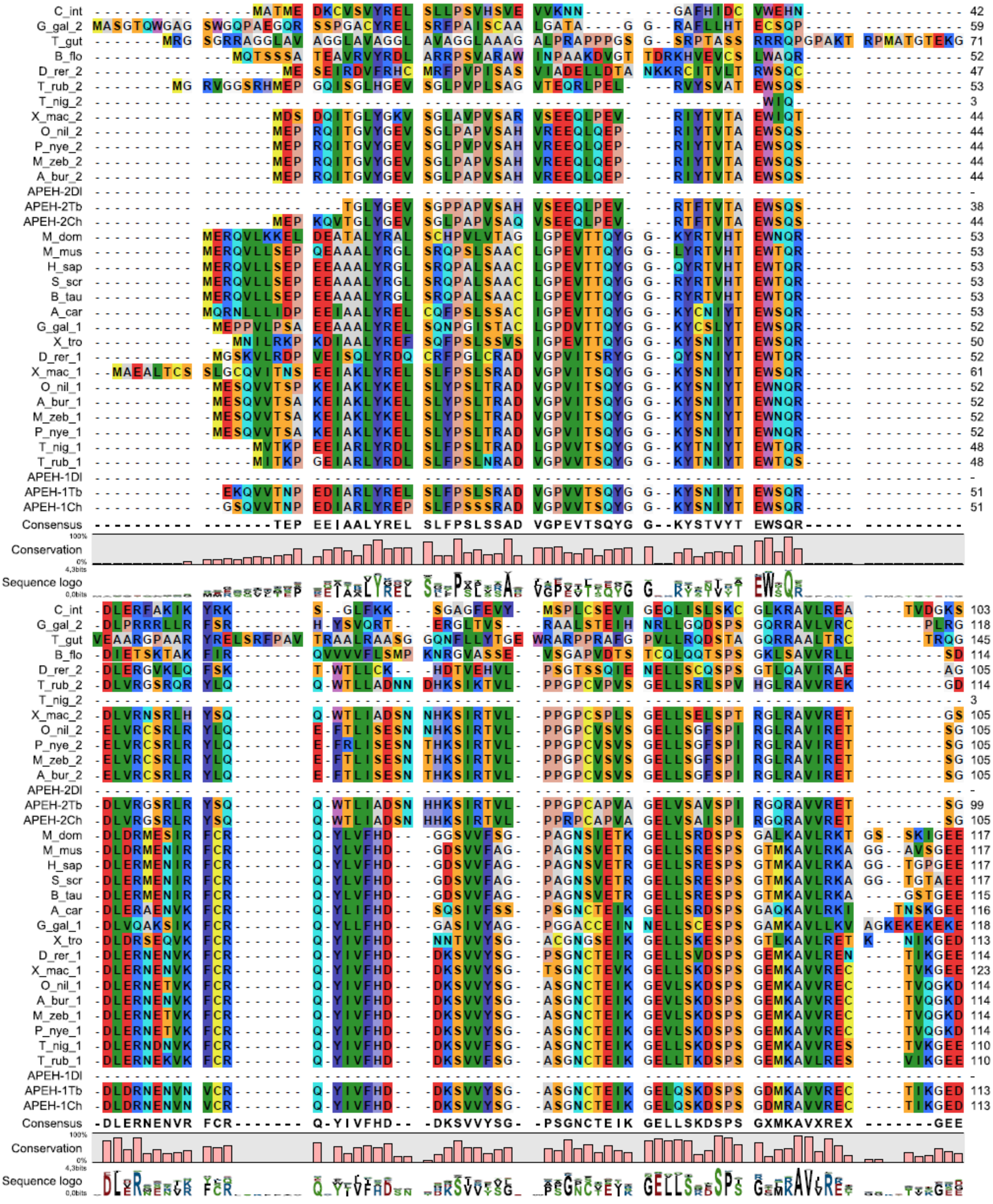

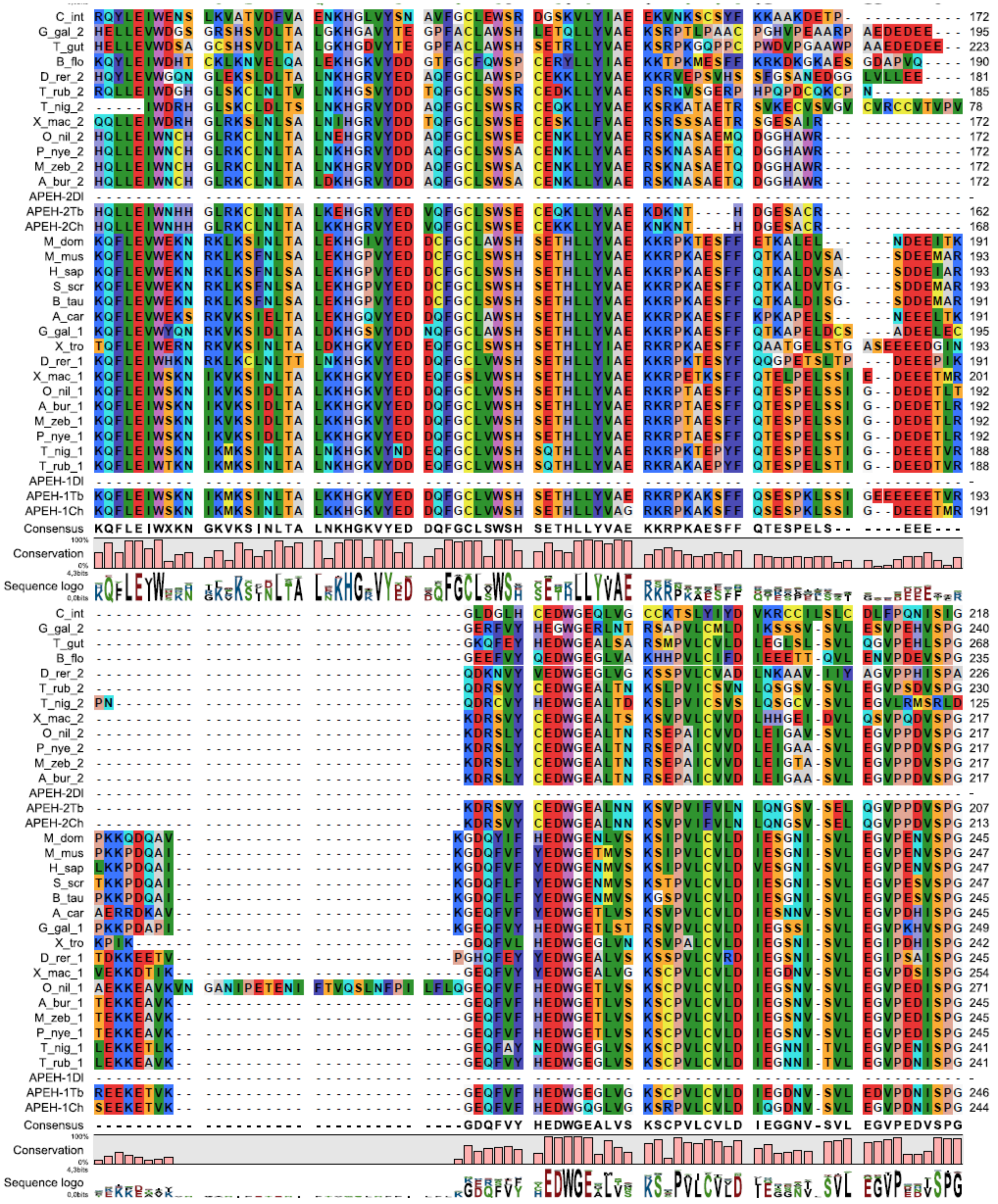
C_int RQYLEIWENS LKVATVDFVA ENKHGLVYSN AVFGCLEWSR DGSKVLYIAE EKVNKSCSYF KKAAKDETP- ---------- 172
G_gal_2 HELLEVWDGS GRSHSVDLTA LGKHGAVYTE GPFACLAWSH LETQLLYVAE KSRPTLPAAC PGHVPEAARP AEDEDEE--- 195
T_gut HELLEVWDSA GCSHSVDLTA LGKHGDVYTE GPFACLAWSH SETRLLYVAE KSRPKGQPPC PWDVPGAAWP AAEDEDEE-- 223
B_flo KQYLEIWDHT CKLKNVELQA LEKHGKVYDD GTFGCFQWSP CERYLLYIAE KKTPKMESFF KRKDKGKAES GDAPVQ---- 190
D_rer_2 HQYLEVWGQN GLEKSLDLTA LNKHGRVYED AQFGCLAWSP CEKKLLYVAE KKRVEPSVHS SFGSANEDGG LVLLEE---- 181
T_rub_2 RQLLEIWDGH GLSKCLNLTV LNKHGSVYDD TQFGCLSWSR CEDKLLYVAE RSRNVSGERP HPQPDCQKCP N--------- 185
T_nig_2 -----IWDRH GLSKCLDLTS LNKHGRVYDD AQFGCLSWSR CEQKLLYVAE KSRKATAETR SVKECVSVGV CVRCCVTVPV 78
X_mac_2 QQLLEIWDRH GLRKSLNLSA LNIHGRVYDD TQFGCLSWSE CESKLLFVAE RSRSSSAETR SGESAIR--- ---------- 172
O_nil_2 HQLLEIWNCH GLRKCLNLTA LNEHGRVYDD AQFGCLSWSE CENKLLYVAE RSKNASAEMQ DGGHAWR--- ---------- 172
P_nye_2 HQLLEIWNCH GLRKCLNLTA LNKHGRVYDD AQFGCLSWSA CENKLLYVAE RSKNASAETQ DGGHAWR--- ---------- 172
M_zeb_2 HQLLEIWNCH GLRKCLNLTA LNKHGRVYDD AQFGCLSWSA CENKLLYVAE RSKNASAETQ DGGHAWR--- ---------- 172
A_bur_2 HQLLEIWNCH GLRKCLNLTA LDKHGRVYDD AQFGCLSWSA CENKLLYVAE RSKNASAETQ DGGHAWR--- ---------- 172
APEH-2Dl ---------- ---------- ---------- ---------- ---------- ---------- ---------- ---------- -
APEH-2Tb HQLLEIWNHH GLRKCLNLTA LKEHGRVYED VQFGCLSWSE CEQKLLYVAE KDKNT----H DGESACR--- ---------- 162
APEH-2Ch HQLLEIWNHH GLRKCLNLTA LKKHGRVYED VQFGCLSWSE CEKKLLYVAE KNKNT----H DGESACR--- ---------- 168
M_dom KQFLEVWEKN RKLKSINLTA LEKHGIVYED DCFGCLAWSH SETHLLYVAE KKRPKTESFF ETKALEL--- ---NDEEITK 191
M_mus KQFLEVWEKN RKLKSFNLSA LEKHGPVYED DCFGCLSWSH SETHLLYVAE KKRPKAESFF QTKALDVSA- ---SDEEMAR 193
H_sap KQFLEVWEKN RKLKSFNLSA LEKHGPVYED DCFGCLSWSH SETHLLYVAE KKRPKAESFF QTKALDVSA- ---SDDEIAR 193
S_scr KQFLEVWEKN RKLKSFNLSA LEKHGPVYED DCFGCLSWSH SETHLLYVAE KKRPKAESFF QTKALDVTG- ---SDDEMAR 193
B_tau KQFLEVWEKN RKLKSFNLSA LEKHGPVYED DCFGCLSWSH SETHLLYVAE KKRPKAESFF QTKALDISG- ---SDDEMAR 191
A_car KQFLEVWEKS RKVKSIELTA LEKHGQVYED DQFGCLAWSH SETHLLYVAE KKRPKAESFF KPKAPELS-- ---NEEELTK 191
G_gal_1 KQFLEVWYQN RKVKSIDLTA LDKHGSVYDD NQFGCLAWSH SETHLLYVAE KKRPKAESFF QTKAPELDCS ---ADEELEC 195
X_tro TQFLEIWERN RKVKSINLTA LDKHGKVYED EQFGCLSWSH SETHLLYIAE RKRPKAESFF QAATGELSTG ASEEEEDGIN 193
D_rer_1 KQFLEIWHKN RKLKCLNLTT LNKHGKVYED DQFGCLVWSH SETHLLYIAE KKRPKTESYF QQGPETSLTP ---DEEEPIK 191
X_mac_1 KQFLEIWSKN IKVKSINLTA LKKHGKVYED EQFGSLVWSH SETHLLYVAE KKRPETKSFF QTELPELSSI E--DEEETMR 201
O_nil_1 KQFLEIWSKN IKVKSIDLTA LKKHGKVYED DQFGCLVWSH SETHLLYVAE RKRPTAESFF QTESPELSSI G--DEDETLT 192
A_bur_1 KQFLEIWSKN IKVKSIDLTA LKKHGKVYED DQFGCLVWSH SETHLLYVAE RKRPTAESFF QTESPELSSI G--DEDETLR 192
M_zeb_1 KQFLEIWSKN IKVKSIDLTA LKKHGKVYED DQFGCLVWSH SETHLLYVAE RKRPTAESFF QTESPELSSI G--DEDETLR 192
P_nye_1 KQFLEIWSKN IKVKSIDLTA LKKHGKVYED DQFGCLVWSH SETHLLYVAE RKRPTAESFF QTESPELSSI G--DEDETLR 192
T_nig_1 KQFLEIWSKN IKMKSINLTA LNKHGKVYND EQFGCLVWSH SQTHLLYVAE RKRPKTEPYF QTESPELSTI G--DEEETVR 188
T_rub_1 KQFLEIWTKN IKMKSINLTA LNKHGKVYDD EQFGCLVWSH SQTHLLYVAE RKRAKAEPYF QTESPELSTI G--DEEDTVR 188
APEH-1Dl ---------- ---------- ---------- ---------- ---------- ---------- ---------- ---------- -
APEH-1Tb KQFLEIWSKN IKMKSINLTA LKKHGKVYED DQFGCLVWSH SETHLLYVAE KKRPKAKSFF QSESPKLSSI GEEEEEETVR 193
APEH-1Ch KQFLEIWSKN IKMKSINLTA LKKHGKVYED DQFGCLVWSH SETHLLYVAG KKRPKAKSFF QSESPKLSSI G--DEEETMR 191
Consensus KQFLEIWXKN GKVKSINLTA LNKHGKVYED DQFGCLSWSH SETHLLYVAE KKRPKAESFF QTESPELS-- ----EEE---
Conservation
Sequence logo
C_int ---------- ---------- ---------- ----GLDGLH CEDWGEQLVG CCKTSLYIYD VKRCCILSLC DLFPQNISIG 218
G_gal_2 ---------- ---------- ---------- ----GERFVY HEGWGERLNT RSAPVLCMLD IKSSSV-SVL ESVPEHVSPG 240
T_gut ---------- ---------- ---------- ----GKQFEY HEDWGEALSA RSMPVLCVLD LEGLSL-SVL QGVPEHLSPG 268
B_flo ---------- ---------- ---------- ----GEEFVY QEDWGEGLVA KHHPVLCIFD IEEETT-QVL ENVPDEVSPG 235
D_rer_2 ---------- ---------- ---------- ----QDKNVY VEDWGEGLVG KSSPVLCVAD LNKAAV-IIY AGVPPHISPA 226
T_rub_2 ---------- ---------- ---------- ----QDRSVY CEDWGEALTN KSLPVICSVN LQSGSV-SVL EGVPSDVSPG 230
T_nig_2 PN-------- ---------- ---------- ----QDRCVY HEDWGEALTD KSLPVICSVS LQSGCV-SVL EGVLRMSRLD 125
X_mac_2 ---------- ---------- ---------- ----KDRSVY CEDWGEALTS KSVPVLCVVD LHHGEI-DVL QSVPQDVSPG 217
O_nil_2 ---------- ---------- ---------- ----KDRSLY CEDWGEALTN RSEPAICVVD LEIGAV-SVL EGVPPDVSPG 217
P_nye_2 ---------- ---------- ---------- ----KDRSLY CEDWGEALTN RSEPAICVVD LEIGAA-SVL EGVPPDVSPG 217
M_zeb_2 ---------- ---------- ---------- ----KDRSLY CEDWGEALTN RSEPAICVVD LEIGTA-SVL EGVPPDVSPG 217
A_bur_2 ---------- ---------- ---------- ----KDRSLY CEDWGEALTN RSEPAICVVD LEIGAA-SVL EGVPPDVSPG 217
APEH-2Dl ---------- ---------- ---------- ---------- ---------- ---------- ---------- ---------- -
APEH-2Tb ---------- ---------- ---------- ----KDRSVY CEDWGEALNN KSVPVIFVLN LQNGSV-SEL QGVPPDVSPG 207
APEH-2Ch ---------- ---------- ---------- ----KDRSVY CEDWGEALNN KSVPVIFVLN LQNGSV-SEL QGVPPDVSPG 213
M_dom PKKQDQAV-- ---------- ---------- ---KGDQYIF HEDWGENLVS KSIPVLCVLD IESGNI-SVL EGVPENVSPG 245
M_mus PKKPDQAI-- ---------- ---------- ---KGDQFVF YEDWGETMVS KSIPVLCVLD IESGNI-SVL EGVPENVSPG 247
H_sap LKKPDQAI-- ---------- ---------- ---KGDQFVF YEDWGENMVS KSIPVLCVLD VESGNI-SVL EGVPENVSPG 247
S_scr TKKPDQAI-- ---------- ---------- ---KGDQFLF YEDWGENMVS KSTPVLCVLD IESGNI-SVL EGVPESVSPG 247
B_tau PKKPDQAI-- ---------- ---------- ---KGDQFLF YEDWGENMVS KGSPVLCVLD IESGNI-SVL EGVPESVSPG 245
A_car AERRDKAV-- ---------- ---------- ---KGEQFVF YEDWGETLVS KSVPVLCVLD IESNNV-SVL EGVPDHISPG 245
G_gal_1 PKKPDAPI-- ---------- ---------- ---KGEQFVY HEDWGETLST RSVPVLCVLD IEGSSI-SVL EGVPKHVSPG 249
X_tro KPIK------ ---------- ---------- ----GDQFVL HEDWGEGLVN KSVPALCVLD IEGSNI-SVL EGIPDHISPG 242
D_rer_1 TDKKEETV-- ---------- ---------- ---PGHQFEY YEDWGEALVS KSSPVLCVRD IEGSNI-SVL EGIPSAISPG 245
X_mac_1 VEKKDTIK-- ---------- ---------- ----GEQFVY YEDWGEALVG KSCPVLCVLD IEGDNV-SVL EGVPDSISPG 254
O_nil_1 AEKKEAVKVN GANIPETENI FTVQSLNFPI LFLQGEQFVF HEDWGETLVS KSCPVLCVLD IEGSNV-SVL EGVPEDISPG 271
A_bur_1 TEKKEAVK-- ---------- ---------- ----GEQFVF HEDWGETLVS KSCPVLCVLD IEGSNV-SVL EGVPEDISPG 245
M_zeb_1 TEKKEAVK-- ---------- ---------- ----GEQFVF HEDWGETLVS KSCPVLCVLD IEGSNV-SVL EGVPEDISPG 245
P_nye_1 TEKKEAVK-- ---------- ---------- ----GEQFVF HEDWGETLVS KSCPVLCVLD IEGSNV-SVL EGVPEDISPG 245
T_nig_1 LEKKETLK-- ---------- ---------- ----GEQFAY NEDWGEGLVS KSCPVLCVLD IEGNNI-TVL EGVPENISPG 241
T_rub_1 LEKKEAVK-- ---------- ---------- ----GEQFVY HEDWGEGLVS KSCPVLCVLD IEGNNI-TVL EGVPDNISPG 241
APEH-1Dl ---------- ---------- ---------- ---------- ---------- ---------- ---------- ---------- -
APEH-1Tb REEKETVK-- ---------- ---------- ----GEQFVF HEDWGEGLVG KSCPVLCVLD IEGDNV-SVL EDVPDNISPG 246
APEH-1Ch SEEKETVK-- ---------- ---------- ----GEQFVF HEDWGQGLVG KSRPVLCVLD IQGDNV-SVL EGVPDNISPG 244
Consensus ---------- ---------- ---------- ----GDQFVY HEDWGEALVS KSCPVLCVLD IEGGNV-SVL EGVPEDVSPG
Conservation
Sequence logo

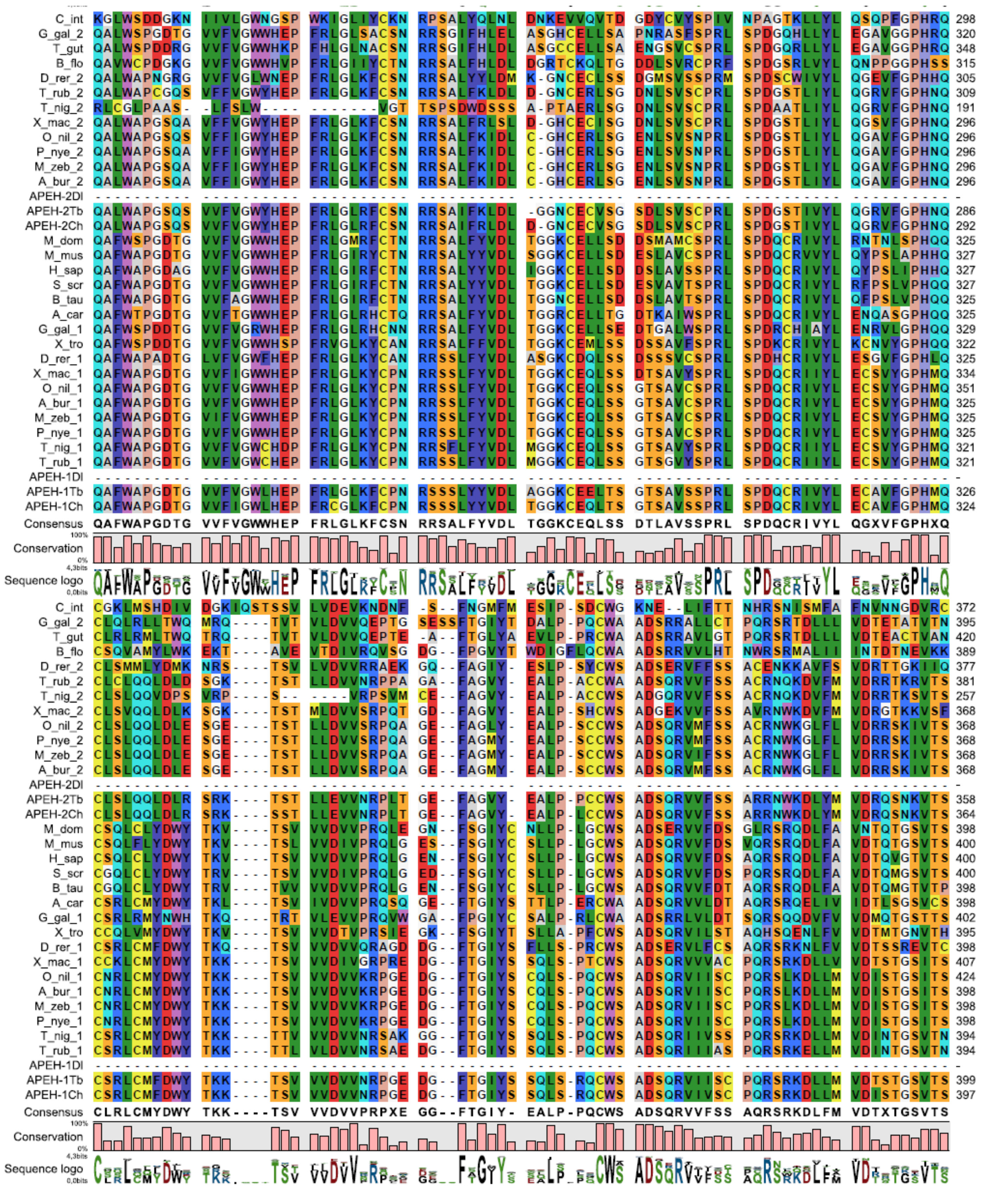

C_int
G_gal_2
T_gut
B_flo
D_rer_2
T_rub_2
T_nig_2
X_mac_2
O_nil_2
P_nye_2
M_zeb_2
A_bur_2
APEH-2Dl
APEH-2Tb
APEH-2Ch
M_dom
M_mus
H_sap
S_scr
B_tau
A_car
G_gal_1
X_tro
D_rer_1
X_mac_1
O_nil_1
A_bur_1
M_zeb_1
P_nye_1
T_nig_1
T_rub_1
APEH-1Dl
APEH-1Tb
APEH-1Ch
Consensus QAFWAPGDTG VVFVGWWHEP FRLGLKFCSN RRSALFYVDL TGGKCEQLSS DTLAVSSPRL SPDQCRIVYL QGXVFGPHXQ
Conservation
Sequence logo
Consensus CLRLCMYDWY TKK----TSV VVDVVPRPXE GG--FTGIY- EALP-PQCWS ADSQRVVFSS AQRSRKDLFM VDTXTGSVTS
Conservation
Sequence logo

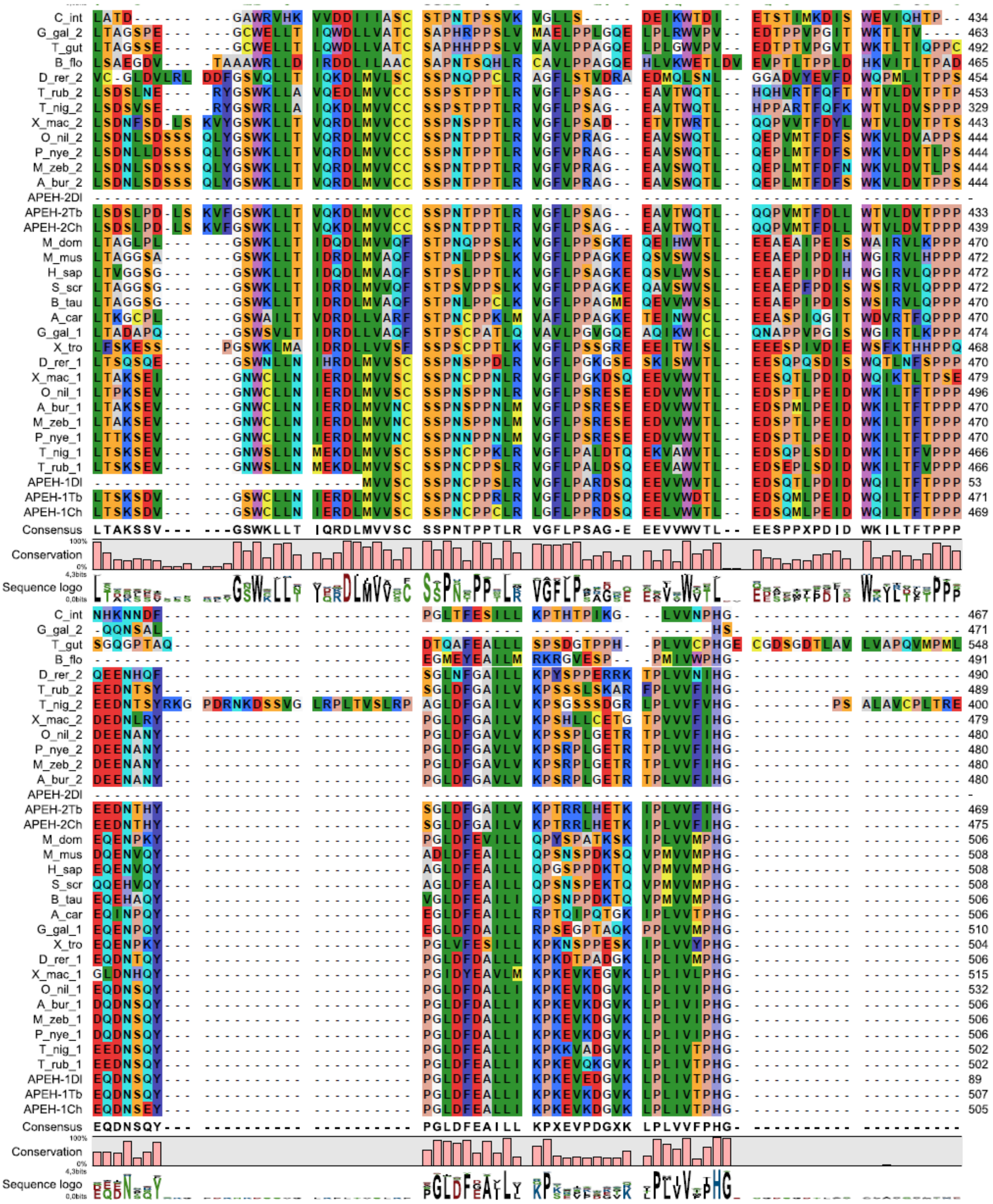

C_int 434
G_gal_2 463
T_gut 492
B_flo 465
D_rer_2 454
T_rub_2 453
T_nig_2 329
X_mac_2 443
O_nil_2 444
P_nye_2 444
M_zeb_2 444
A_bur_2 444
APEH-2Dl
APEH-2Tb 433
APEH-2Ch 439
M_dom 470
M_mus 472
H_sap 472
S_scr 472
B_tau 470
A_car 470
G_gal_1 474
X_tro 468
D_rer_1 470
X_mac_1 479
O_nil_1 496
A_bur_1 470
M_zeb_1 470
P_nye_1 470
T_nig_1 466
T_rub_1 466
APEH-1Dl 53
APEH-1Tb 471
APEH-1Ch 469
Consensus LTAKSSV--- ---GSWKLLT IQRDLMVVSC SSPNTPPTLR VGFLPSAG-E EEVVWVTL-- EESPPXPDID WKILTFTPPP
100%
Conservation
0%
4,3bits
Sequence logo
0,0bits
C_int 467
G_gal_2 471
T_gut 548
B_flo 491
D_rer_2 490
T_rub_2 489
T_nig_2 400
X_mac_2 479
O_nil_2 480
P_nye_2 480
M_zeb_2 480
A_bur_2 480
APEH-2Dl
APEH-2Tb 469
APEH-2Ch 475
M_dom 506
M_mus 508
H_sap 508
S_scr 508
B_tau 506
A_car 506
G_gal_1 510
X_tro 504
D_rer_1 506
X_mac_1 515
O_nil_1 532
A_bur_1 506
M_zeb_1 506
P_nye_1 506
T_nig_1 502
T_rub_1 502
APEH-1Dl 89
APEH-1Tb 507
APEH-1Ch 505
Consensus EQDNSQY--- ---------- ---------- PGLDFEAILL KPXEVPDGXK LPLVVFPHG- ---------- ----------
100%
Conservation
0%
4,3bits
Sequence logo
0,0bits

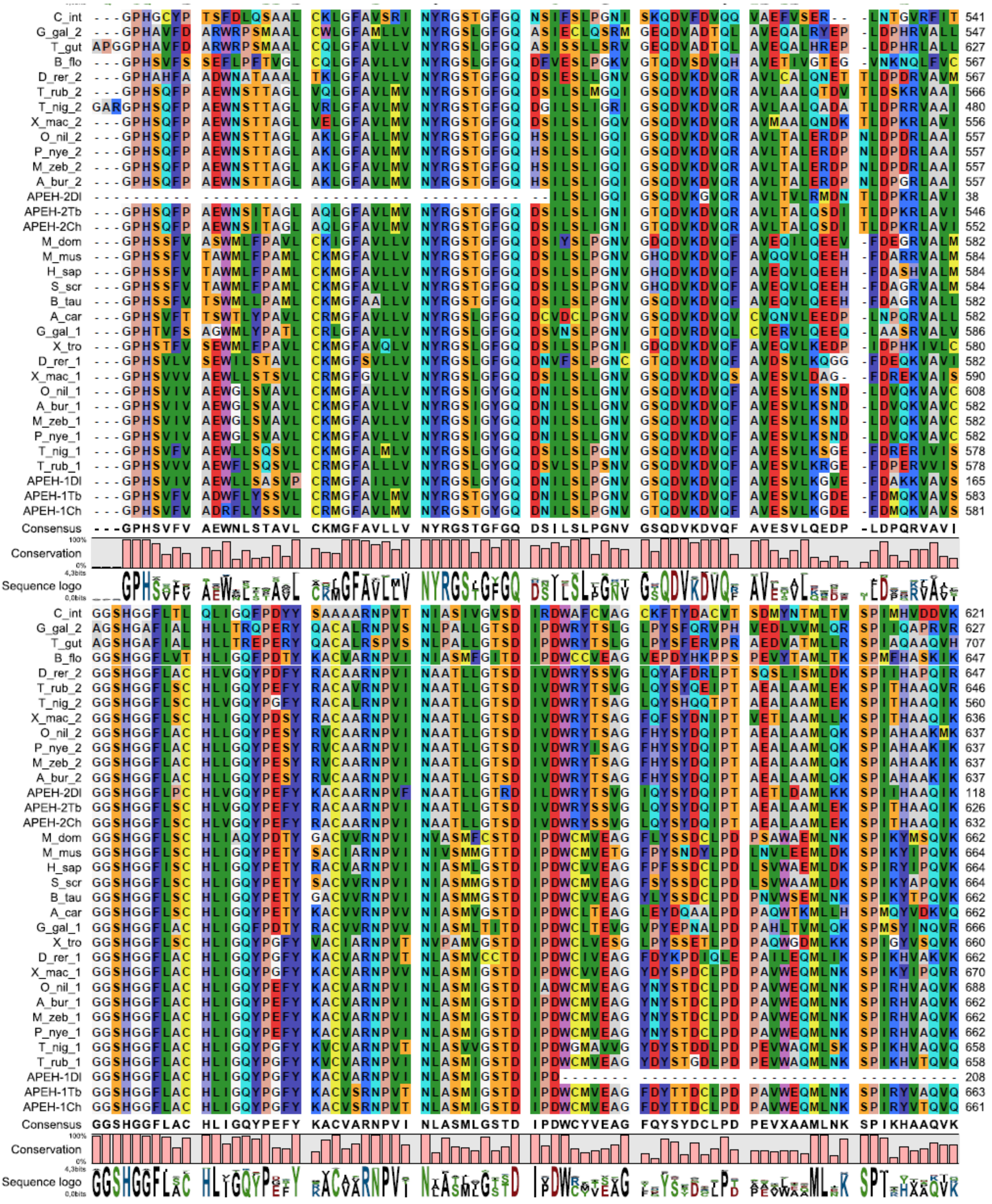
C_int
G_gal_2
T_gut
B_flo
D_rer_2
T_rub_2
T_nig_2
X_mac_2
O_nil_2
P_nye_2
M_zeb_2
A_bur_2
APEH-2DI
APEH-2Tb
APEH-2Ch
M_dom
M_mus
H_sap
S_scr
B_tau
A_car
G_gal_1
X_tro
D_rer_1
X_mac_1
O_nil_1
A_bur_1
M_zeb_1
P_nye_1
T_nig_1
T_rub_1
APEH-1DI
APEH-1Tb
APEH-1Ch
Consensus
---GPHSVFV AEWNLSTAVL CKMGFAVLLV NYRGSTGFGQ DSILSLPGNV GSQDVKDVQF AVESVLQEDP -LDPQRVAVI
Conservation
100%
0%
Sequence logo
4,3bits
0,0bits
Consensus
GGSHGGFLAC HLIGQYPEFY KACVARNPVI NLASMLGSTD IPDWCYVEAG FQYSYDCLPD PEVXAAMLNK SPIKHAAQVK
Conservation
Sequence logo

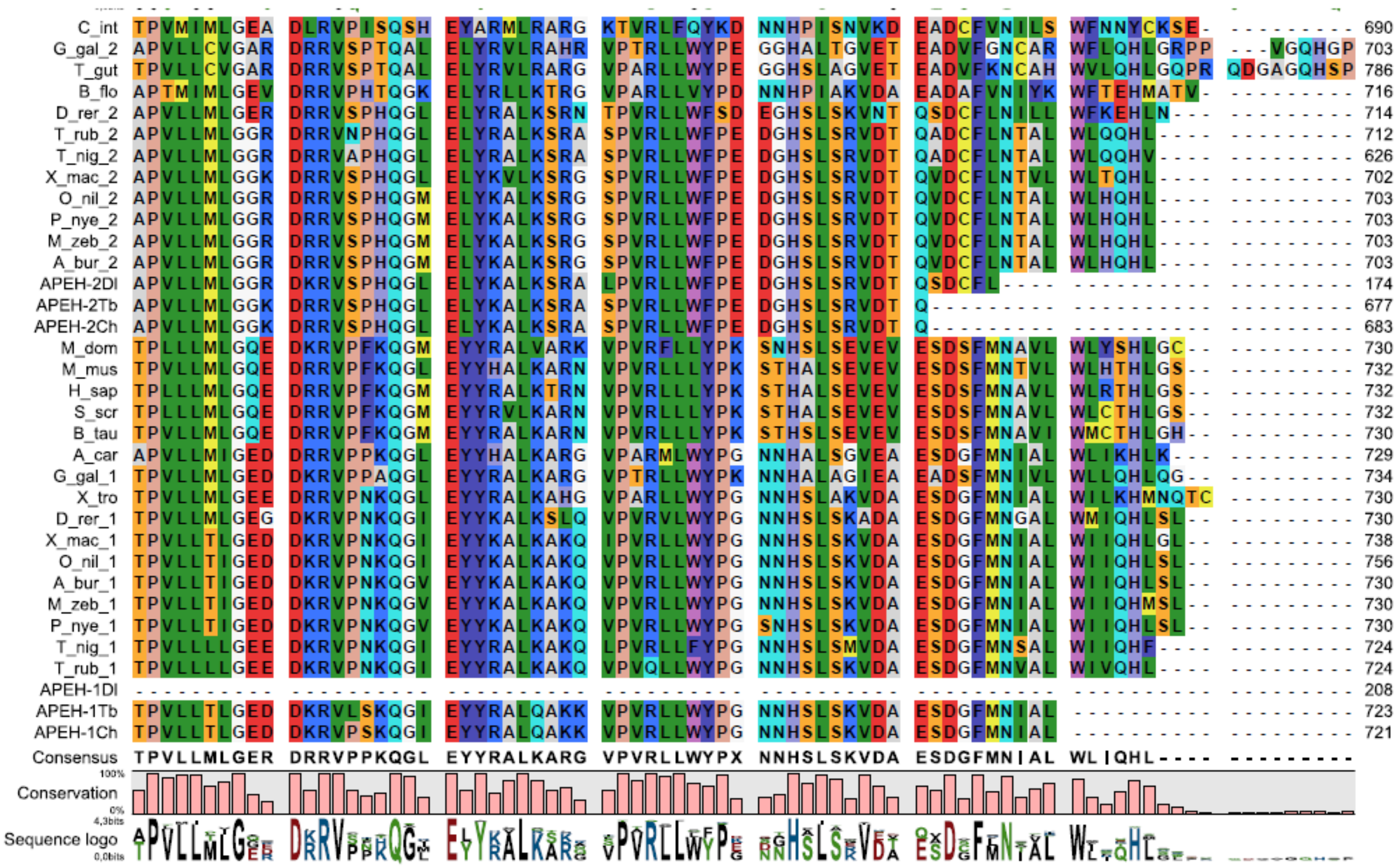

Figure S3. Muscle alignment of APEH-1 and APEH-2 sequences from different sources. The consensus sequence, the conservation histogram and the sequence logo are shown at the bottom of the alignment
