# Supplementary material for: A New APEH Cluster with Antioxidant Functions in the Antarctic Hemoglobinless Icefish Chionodraco hamatus
Source: PLoS One. 2015 May 6;10(5):e0125594. doi: 10.1371/journal.pone.0125594 (PMC4422685; doi:10.1371/journal.pone.0125594)
Supplement: S4 Fig — Purified APEH-1 and APEH-2 from blood cells of both fish and the commercially available APEH from porcine liver (APEHpl) were loaded as controls. All the experiments were performed in duplicate on two different protein preparations. (PDF) [file pone.0125594.s004.pdf]

APEH isoforms tissue distribution

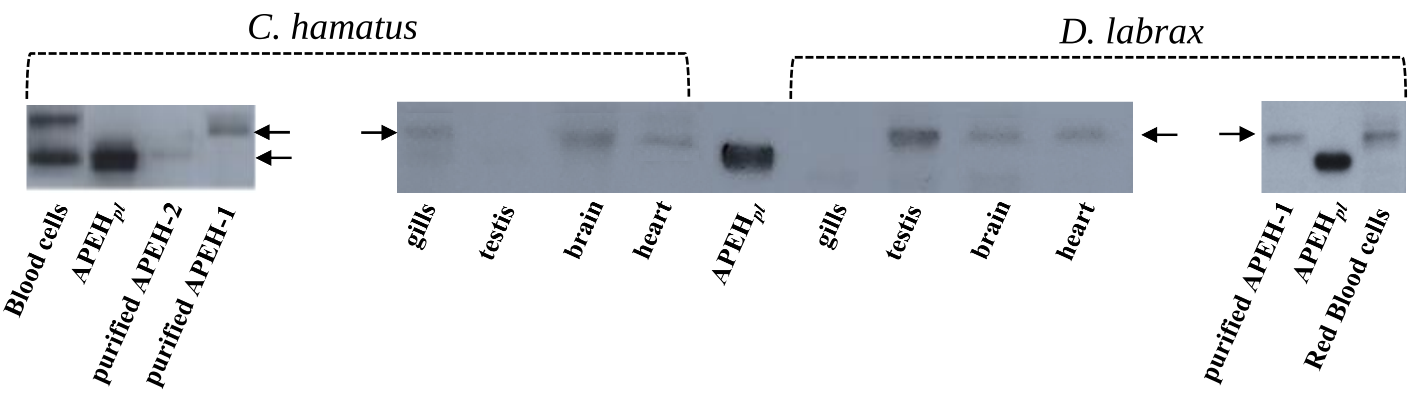

Figure S4. Western blot analysis of APEH-1 and APEH-2 isoforms in blood cells and different tissues from *C. hamatus* and *D. labrax*. Purified APEH-1 and APEH-2 from blood cells of both fish and the commercially available APEH from porcine liver ($APEH_{pl}$) were loaded as controls. All the experiments were performed in duplicate on two different protein preparations.
