# Supplementary material for: A New APEH Cluster with Antioxidant Functions in the Antarctic Hemoglobinless Icefish Chionodraco hamatus
Source: PLoS One. 2015 May 6;10(5):e0125594. doi: 10.1371/journal.pone.0125594 (PMC4422685; doi:10.1371/journal.pone.0125594)
Supplement: S1 Table — (PDF) [file pone.0125594.s005.pdf]

Table S1. APEH chains utilized for phylogenetic analysis

chain	Source organism	Accession number
H_sap	*Homo sapiens*	NP_001631
M_mus	*Mus musculus*	NP_666338
S_scr	*Sus scrofa*	AAB36056
B_tau	*Bos taurus*	XP_871965
M_dom	*Monodelphis domestica*	XP_001378111
A_car	*Anolis carolinensis*	XP_003217605
G_gal_1	*Gallus gallus*	XP_414270
G_gal_2	*Gallus gallus*	XP_001234120
T_gut	*Taeniopygia guttata*	XP_002190622
X_tro	*Xenopus (Silurana) tropicalis*	XP_002936520
D_rer_1	*Danio rerio*	NP_942570
D_rer_2	*Danio rerio*	NP_001035437
T_nig_1	Tetraodon nigroviridis	CAG07332
T_nig_2	Tetraodon nigroviridis	CAF93359
T_rub_1	*Takifugu rubripes*	XP_003963327
T_rub_2	*Takifugu rubripes*	XP_003963075
X_mac_1	*Xiphophorus maculatus*	XP_005811291
X_mac_2	*Xiphophorus maculatus*	XP_005800228
O_nil_1	*Oreochromis niloticus*	XP_003448339
O_nil_2	*Oreochromis niloticus*	XP_003444889
A_bur_1	*Astatotilapia burtoni*	XP_005914685
A_bur_2	*Astatotilapia burtoni*	XP_005937909

M_zeb_1	*Maylandia zebra*	XP_004558946
M_zeb_2	*Maylandia zebra*	XP_004554076
P_nye_1	*Pundamilia nyererei*	XP_005732521
P_nye_2	*Pundamilia nyererei*	XP_005730746
C_int	*Ciona intestinalis*	XP_002121171
B_flo	*Branchiostoma floridae*	XP_002611296
APEH-1$_{Tb}$	*Trematomus bernacchii*	KC626077
APEH-2$_{Tb}$	*Trematomus bernacchii*	KC626078
**APEH-1$_{Ch}$**	*Chionodraco hamatus*	this paper
**APEH-2$_{Ch}$**	*Chionodraco hamatus*	this paper
**APEH-1$_{Dl}$**	*Dicentrachus labrax*	this paper
**APEH-2$_{Dl}$**	*Dicentrachus labrax*	this paper
