# Supplementary material for: A New APEH Cluster with Antioxidant Functions in the Antarctic Hemoglobinless Icefish Chionodraco hamatus
Source: PLoS One. 2015 May 6;10(5):e0125594. doi: 10.1371/journal.pone.0125594 (PMC4422685; doi:10.1371/journal.pone.0125594)
Supplement: S2 Table — (PDF) [file pone.0125594.s006.pdf]

Table S2

A) Purification of APEH-1$_{Dl}$ from *Dicentrarchus labrax*

Purification step	Total activity (U)	Total protein (mg)	Specific activity (U/mg)	Purification fold	Yield (%)
Hemolysate	4.43×10^6	314.50	1.40×10^4	1	100
DEAE	2.26×10^6	10.70	2.11×10^5	15	51
Hitrap Phenyl	1.33×10^6	3.80	3.50×10^5	25	30
Superdex 200	7.53×10^5	0.90	8.36×10^5	59	17

B) Purification of APEH-1$_{Ch}$ and APEH-2$_{Ch}$ from *Chionodraco hamatus*

Purification step	Total activity (U)	Total protein (mg)	Specific activity (U/mg)	Purification fold	Yield (%)
Hemolysate	2.70×10^4	22	1.23×10^3	1	100
DEAE APEH-1	1.74×10^4	2	8.68×10^3	7	64
DEAE APEH-2	9.46×10^3	0.14	6.75×10^4	55	35
Mono S APEH-1	1.70×10^4	0.05	3.40×10^5	278	63
Mono S APEH-2	7.00×10^3	0.018	3.89×10^5	317	26
Phenyl APEH-1	1.55×10^4	0.014	1.11×10^6	902	57
Sup200 APEH-2	2.52×10^3	0.009	2.80×10^5	228	10
