# Supplementary material for: A New APEH Cluster with Antioxidant Functions in the Antarctic Hemoglobinless Icefish Chionodraco hamatus
Source: PLoS One. 2015 May 6;10(5):e0125594. doi: 10.1371/journal.pone.0125594 (PMC4422685; doi:10.1371/journal.pone.0125594)
Supplement: S3 Table — Side chain atoms within 3 Angstroms from the catalytic triade in APEH-1Ch (i.e. Ser585, Asp673, His705) and APEH-2Ch (i.e. Ser555, Asp643, His675) are listed. The column with notes indicate when amino acid is part of the catalytic triade, and when atoms from side chains are charged. (PDF) [file pone.0125594.s007.pdf]

Table S3. Catalytic site environment for APEH-1$_{Ch}$ and APEH-2$_{Ch}$. Side chain atoms within 3 Angstroms from the catalytic triade in APEH-1$_{Ch}$ (i.e. Ser585, Asp673, His705) and APEH-2$_{Ch}$ (i.e. Ser555, Asp643, His675) are listed. The column with notes indicate when amino acid is part of the catalytic triade, and when atoms from side chains are charged.

APEH-1$_{Ch}$

Amino acid	Atoms	Notes
VAL 141	CA,CB,CG2,C	
GLU 143	CG,CD,OE1	negative charge
ASP 145	OD2	negative charge
GLN 246	N,CA,CB,NE2,C,O	
SER 585	N,CA,CB,OG,C,O	catalytic triade
HIS 586	N,CA,C	
ASN 609	CG,OD1	
ASP 673	N,CA,CB,CG,OD1,OD2,C,O	catalytic triade
VAL 676	N,CG2,C,O	
ASN 704	CA,CB,ND2,C,O	
HIS 705	N,CA,ND1,CG,CB,NE2,CD2,CE1,C,O	catalytic triade

APEH-2$_{Ch}$

Amino acid	Atoms	Notes
VAL 132	N,CA,CB,CG1,CG2,O	
GLU 134	CG	
GLN 214	N,CA,CB,C,O	
SER 555	N,CA,CB,OG,C,O	catalytic triade
ASN 579	ND2	
ASP 643	N,CA,CB,CG,OD1,OD2,C,O	catalytic triade
ARG 645	CZ,NH1,NH2	positive charge
HIS 675	N,CA,ND1,CG,CB,NE2,CD2,CE1,C,O	catalytic triade
